# Correction: Effects of chronic exposure to arsenic on the fecal carriage of antibiotic-resistant *Escherichia coli* among people in rural Bangladesh

**DOI:** 10.1371/journal.ppat.1011690

**Published:** 2023-09-29

**Authors:** Mohammed Badrul Amin, Prabhat Kumar Talukdar, Muhammad Asaduzzaman, Subarna Roy, Brandon M. Flatgard, Md. Rayhanul Islam, Sumita Rani Saha, Yushuf Sharker, Zahid Hayat Mahmud, Tala Navab-Daneshmand, Molly L. Kile, Karen Levy, Timothy R. Julian, Mohammad Aminul Islam

## Notice of Republication

This article was republished on September 12, 2023 to add Yushuf Sharker as the eighth author. The author contributions and Acknowledgments in the original publication did not correctly indicate Yushuf Sharker’s contributions. The About the Authors section was updated accordingly. Please download this article again to view the correct version. The originally published, uncorrected article and the republished, corrected article are provided here for reference.

## Supporting information

S1 File Originally published, uncorrected article. (PDF)

S2 File Republished, corrected article. (PDF)
