# Supplementary material for: Effects of chronic exposure to arsenic on the fecal carriage of antibiotic-resistant Escherichia coli among people in rural Bangladesh
Source: PLoS Pathog. 2022 Dec 8;18(12):e1010952. doi: 10.1371/journal.ppat.1010952 (PMC9731454; doi:10.1371/journal.ppat.1010952)
Supplement: S1 Table — (DOCX) [file ppat.1010952.s001.docx]

**S1 Table. Arsenic concentration in water samples from Hajiganj and Matlab areas.**

| Hajiganj area | | Matlab area | |
| --- | --- | --- | --- |
| Sample ID | Arsenic concentration (µg/L) | Sample ID | Arsenic concentration (µg/L) |
| RH-WU-001 | 458 | RM-WU-001 | 1 |
| RH-WU-002 | 508 | RM-WU-002 | 0 |
| RH-WU-003 | 502 | RM-WU-003 | 0 |
| RH-WU-004 | 506 | RM-WU-004 | 1 |
| RH-WU-005 | 463 | RM-WU-005 | 0 |
| RH-WU-006 | 521 | RM-WU-006 | 0 |
| RH-WU-007 | 549 | RM-WU-007 | 3 |
| RH-WU-008 | 492 | RM-WU-008 | 0 |
| RH-WU-009 | 553 | RM-WU-009 | 1 |
| RH-WU-010 | 479 | RM-WU-010 | 0 |
| RH-WU-011 | 465 | RM-WU-011 | 0 |
| RH-WU-012 | 600 | RM-WU-012 | 1 |
| RH-WU-013 | 508 | RM-WU-013 | 0 |
| RH-WU-014 | 507 | RM-WU-014 | 1 |
| RH-WU-015 | 536 | RM-WU-015 | 1 |
| RH-WU-016 | 511 | RM-WU-016 | 2 |
| RH-WU-017 | 519 | RM-WU-017 | 0 |
| RH-WU-018 | 542 | RM-WU-018 | 0 |
| RH-WU-019 | 446 | RM-WU-019 | 3 |
| RH-WU-020 | 469 | RM-WU-020 | 2 |
| RH-WU-021 | 400 | RM-WU-021 | 1 |
| RH-WU-022 | 618 | RM-WU-022 | 0 |
| RH-WU-023 | 402 | RM-WU-023 | 0 |
| RH-WU-024 | 421 | RM-WU-024 | 0 |
| RH-WU-025 | 417 | RM-WU-025 | 1 |
| RH-WU-026 | 729 | RM-WU-026 | 1 |
| RH-WU-027 | 387 | RM-WU-027 | 0 |
| RH-WU-028 | 639 | RM-WU-028 | 20 |
| RH-WU-029 | 635 | RM-WU-029 | 0 |
| RH-WU-030 | 355 | RM-WU-030 | 0 |
| RH-WU-031 | 349 | RM-WU-031 | 1 |
| RH-WU-032 | 356 | RM-WU-032 | 0 |
| RH-WU-033 | 223 | RM-WU-033 | 0 |
| RH-WU-034 | 293 | RM-WU-034 | 0 |
| RH-WU-035 | 333 | RM-WU-035 | 0 |
| RH-WU-036 | 545 | RM-WU-036 | 0 |
| RH-WU-037 | 485 | RM-WU-037 | 1 |
| RH-WU-038 | 495 | RM-WU-038 | 0 |
| RH-WU-039 | 249 | RM-WU-039 | 0 |
| RH-WU-040 | 393 | RM-WU-040 | 5 |
| RH-WU-041 | 440 | RM-WU-041 | 2 |
| RH-WU-042 | 527 | RM-WU-042 | 0 |
| RH-WU-043 | 313 | RM-WU-043 | 1 |
| RH-WU-044 | 348 | RM-WU-044 | 1 |
| RH-WU-045 | 373 | RM-WU-045 | 1 |
| RH-WU-046 | 427 | RM-WU-046 | 1 |
| RH-WU-047 | 369 | RM-WU-047 | 0 |
| RH-WU-048 | 483 | RM-WU-048 | 0 |
| RH-WU-049 | 530 | RM-WU-049 | 1 |
| RH-WU-050 | 561 | RM-WU-050 | 0 |
