# Supplementary material for: Effects of chronic exposure to arsenic on the fecal carriage of antibiotic-resistant Escherichia coli among people in rural Bangladesh
Source: PLoS Pathog. 2022 Dec 8;18(12):e1010952. doi: 10.1371/journal.ppat.1010952 (PMC9731454; doi:10.1371/journal.ppat.1010952)
Supplement: S2 Table — (DOCX) [file ppat.1010952.s002.docx]

**S2 Table. PCR primers used in this study to identify arsenic resistance genes.**

| **Target Gene** | **Oligo Name** | **Sequences (5' -3')** | **References** |
| --- | --- | --- | --- |
| *Chromosomal and plasmid* | | | |
| *arsA* | *arsA*-F | TCCTGGATTGTCGGCTCTTG | [1] |
| *arsA* | *arsA*-R | ATCTGTCAGTAATCCGGTAA | [1] |
| *arsB* | *arsB*-F | CGGTGGTGTGGAATATTGTC | [1] |
| *arsB* | *arsB*-R | GTCAGAATAAGAGCCGCACC | [1] |
| *arsC* | *arsC*-F | GTAATACGCTGGAGATGATCCG | [1] |
| *arsC* | *arsC*-R | TTTTCCTGCTTCATCAACGAC | [1] |
| *Chromosomal* |  |  |  |
| *arsR* | K12_*arsR*-F | CGTCTGGGCATCGTTTTACT | This study |
| *arsR* | K12_*arsR*-R | TGCTGGAATATGCGGTGATA | This study |
| *Plasmid* | | | |
| *arsD* | R46_*arsD*-F | TGTTTGATCCGGCGATGT | This study |
| *arsD* | R46_*arsD*-R | TTCCAGCGGTATACCAAACC | This study |
| *arsR* | R46_*arsR*-F | GCTACTGCTCAGGGAGATGG | This study |
| *arsR* | R46_*arsR*-R | CTGTACGTCGTCCTGTTGAC | This study |

**Reference**

1. Saltikov CW, Olson BH. Homology of *Escherichia coli* R773 *arsA*, *arsB*, and *arsC* genes in arsenic-resistant bacteria isolated from raw sewage and arsenic-enriched creek waters. Appl Environ Microbiol. 2002;68(1):280-8. Epub 2002/01/05. doi: 10.1128/AEM.68.1.280-288.2002. PubMed PMID: 11772637; PubMed Central PMCID: PMCPMC126541.
