# Supplementary figures and images for: Effects of chronic exposure to arsenic on the fecal carriage of antibiotic-resistant Escherichia coli among people in rural Bangladesh
Source: PLoS Pathog. 2022 Dec 8;18(12):e1010952. doi: 10.1371/journal.ppat.1010952 (PMC9731454; doi:10.1371/journal.ppat.1010952)

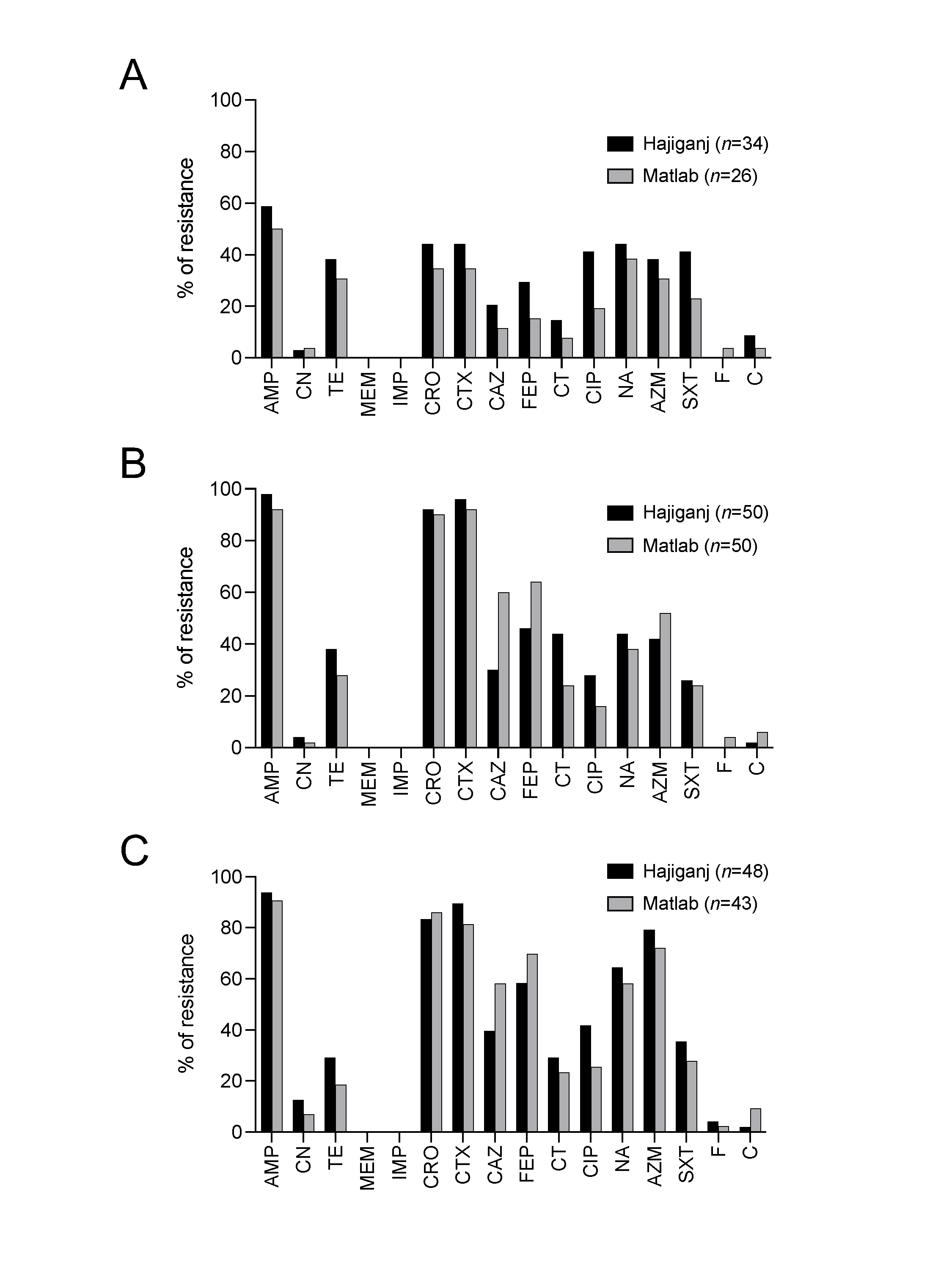

Supplement: S1 Fig — Antibiotic resistance of all E. coli isolates in A) drinking water, B) mother stool, and C) child stool samples collected from Hajiganj (high arsenic exposure) and Matlab (low arsenic exposure) was determined against 16 commercially available antibiotics as described in the Methods section. AMP, ampicillin; CN, gentamycin; TE, tetracycline; MEM, meropenem; IMP, imipenem; CRO, ceftriaxone, CTX, cefotaxime; CAZ, ceftazidime; FEP, cefepime; CT, colistin; CIP, ciprofloxacin; NA, nalidixic acid; AZM, azithromycin; SXT, trimethoprim-sulfamethoxazole; F, nitrofurantoin; C, chloramphenicol. ‘n’ indicates the number of E. coli isolates. (TIFF) [file ppat.1010952.s003.tiff]
