# Supplementary material for: Correction: Effects of chronic exposure to arsenic on the fecal carriage of antibiotic-resistant Escherichia coli among people in rural Bangladesh
Source: PLoS Pathog. 2023 Sep 29;19(9):e1011690. doi: 10.1371/journal.ppat.1011690 (PMC10540963; doi:10.1371/journal.ppat.1011690)
Supplement: S2 File — (PDF) [file ppat.1011690.s002.pdf]

PLOS PATHOGENS

RESEARCH ARTICLE

Effects of chronic exposure to arsenic on the fecal carriage of antibiotic-resistant *Escherichia coli* among people in rural Bangladesh

Mohammed Badrul Amin[1☯], Prabhat Kumar Talukdar[2☯], Muhammad Asaduzzaman[1,3], Subarna Roy[1], Brandon M. Flatgard[2], Md. Rayhanul Islam[1], Sumita Rani Saha[1], Yushuf Sharker[4], Zahid Hayat Mahmud[5], Tala Navab-Daneshmand[6], Molly L. Kile[7], Karen Levy[8], Timothy R. Julian[9], Mohammad Aminul Islam[1,2]*[iD]

1 Laboratory of Food Safety and One Health, Laboratory Sciences and Services Division, International Centre for Diarrhoeal Disease Research, Bangladesh (icddr,b), Dhaka, Bangladesh, **2** Paul G. Allen School for Global Health, Washington State University, Pullman, Washington, United States of America, **3** Department of Community Medicine and Global Health, Institute of Health and Society, Faculty of Medicine, University of Oslo, Oslo, Norway, **4** Center for Data Research and Analytics LLC, Bethesda, Maryland, United States of America, **5** Laboratory of Environmental Health, Laboratory Sciences and Services Division, International Centre for Diarrhoeal Disease Research, Bangladesh (icddr,b), Dhaka, Bangladesh, **6** School of Chemical, Biological, and Environmental Engineering, Oregon State University, Corvallis, Oregon, United States of America, **7** School of Biological and Population Health Sciences, Oregon State University, Corvallis, Oregon, United States of America, **8** Department of Environmental and Occupational Health Sciences, University of Washington, Washington, United States of America, **9** Eawag, Swiss Federal Institute of Aquatic Science and Technology, Dübendorf, Switzerland

☯ These authors contributed equally to this work.
* amin.islam@wsu.edu

Check for updates

OPEN ACCESS

Citation: Amin MB, Talukdar PK, Asaduzzaman M, Roy S, Flatgard BM, Islam M.R, et al. (2022) Effects of chronic exposure to arsenic on the fecal carriage of antibiotic-resistant *Escherichia coli* among people in rural Bangladesh. PLoS Pathog 18(12): e1010952. https://doi.org/10.1371/journal.ppat.1010952

Editor: Eric Oswald, INSERM U1220, FRANCE

Received: July 27, 2022

Accepted: October 26, 2022

Published: December 8, 2022

Copyright: © 2022 Amin et al. This is an open access article distributed under the terms of the Creative Commons Attribution License, which permits unrestricted use, distribution, and reproduction in any medium, provided the original author and source are credited.

Data Availability Statement: The whole genome sequencing (WGS) of 30 *E. coli* isolates was reported previously and the data are available in GenBank under accession numbers CP050193-CP050222. All other relevant data are within the manuscript and its Supporting Information files.

Funding: The study was supported by a REACH catalyst grant, United Kingdom to icddr,b (icddr,b Grant No. GR-01507) in which MAI was the Project Lead, and Paul G. Allen School for Global Health, Washington State University (Startup grant:

Abstract

Antibiotic resistance is a leading cause of hospitalization and death worldwide. Heavy metals such as arsenic have been shown to drive co-selection of antibiotic resistance, suggesting arsenic-contaminated drinking water is a risk factor for antibiotic resistance carriage. This study aimed to determine the prevalence and abundance of antibiotic-resistant *Escherichia coli* (AR-Ec) among people and drinking water in high (Hajiganj, >100 μg/L) and low arsenic-contaminated (Matlab, <20 μg/L) areas in Bangladesh. Drinking water and stool from mothers and their children (<1 year) were collected from 50 households per area. AR-Ec was detected via selective culture plating and isolates were tested for antibiotic resistance, arsenic resistance, and diarrheagenic genes by PCR. Whole-genome sequencing (WGS) analysis was done for 30 *E. coli* isolates from 10 households. Prevalence of AR-Ec was significantly higher in water in Hajiganj (48%) compared to water in Matlab (22%, $p < 0.05$) and among children in Hajiganj (94%) compared to children in Matlab (76%, $p < 0.05$), but not among mothers. A significantly higher proportion of *E. coli* isolates from Hajiganj were multidrug-resistant (83%) compared to isolates from Matlab (71%, $p < 0.05$). Co-resistance to arsenic and multiple antibiotics (MAR index >0.2) was observed in a higher proportion of water (78%) and child stool (100%) isolates in Hajiganj than in water (57%) and children (89%) in Matlab ($p < 0.05$). The odds of arsenic-resistant bacteria being resistant to third-generation cephalosporin antibiotics were higher compared to arsenic-sensitive

PG00005723 to MAI). The funders had no role in study design, data collection and analysis, decision to publish, or preparation of the manuscript.

Competing interests: The authors have declared that no competing interests exist.

bacteria (odds ratios, OR 1.2–7.0, p <0.01). WGS-based phylogenetic analysis of *E. coli* isolates did not reveal any clustering based on arsenic exposure and no significant difference in resistome was found among the isolates between the two areas. The positive association detected between arsenic exposure and antibiotic resistance carriage among children in arsenic-affected areas in Bangladesh is an important public health concern that warrants redoubling efforts to reduce arsenic exposure.

Author summary

Antibiotic resistance is one of the leading causes of death and hospitalization worldwide. While the major drivers of antibiotic resistance are the overuse and misuse of antibiotics, natural elements such as heavy metals can also promote antibiotic resistance. In this observational study, we investigated the prevalence and abundance of antibiotic-resistant bacteria in drinking water samples and among mothers and children from the same households in two rural areas of Bangladesh with high and low levels of arsenic contamination in drinking water. We found that the prevalence of antibiotic-resistant *E. coli* was significantly higher in water and among children in high arsenic-contaminated areas compared to the low arsenic-contaminated areas. We also found that a higher proportion of *E. coli* isolates from the high arsenic area were resistant to multiple antibiotics including penicillin, cephalosporin, and chloramphenicol. Arsenic-resistant bacteria are more likely to be resistant to certain groups of antibiotics, including third-generation cephalosporins. Overall, this study suggests that arsenic exposure could be an important risk factor for the carriage of antibiotic-resistant organisms which needs to be taken into consideration when designing community-based interventions to combat antimicrobial resistance.

Introduction

Antimicrobial resistance (AMR) is one of the leading threats to public health in the 21st century. The number of hospitalizations and deaths due to AMR infections has been increasing in recent years. In 2019, globally there were an estimated 4.95 million deaths associated with bacterial AMR, including 1.27 million deaths attributable to bacterial AMR [1]. The emergence and spread of AMR infections are more common in resource-poor countries due to the inappropriate use of antibiotics in healthcare, agriculture, and community settings. Bangladesh is known as a hotspot of AMR, with numerous reports showing the widespread prevalence of antibiotic-resistant organisms (ARO) in humans, food-producing animals, wild animals, and the environment including drinking water, wastewater, surface water, and courtyard soil [2–10].

AMR develops when microorganisms are exposed to sub-lethal concentrations of antibiotics and/or antimicrobial agents [11]. In addition to anthropogenic drivers, there are lesser-known natural elements that contribute to the emergence and dissemination of AMR in certain geographic locations. Heavy metals such as arsenic, cadmium, lead, and chromium can function as antimicrobials, and exposure to these heavy metals can co-select resistance to antibiotics [12–16]. Heavy metals are also more stable and can continue to exert selective pressure on bacteria over a longer period than antibiotic residues [17,18], which decrease in the environment due to degradation, absorption, and sequestration over time [19].

Arsenic is a toxic metalloid ubiquitously present either in inorganic or organic forms in the environment (soil, air, and water). The toxicological effects of arsenic depend on its chemical

form and oxidation state. Arsenite is the most toxic form and is commonly detected in water, soil, and food [20]. High concentrations of arsenic in groundwater and soil have been an issue that impacts human health in many parts of the world, particularly in the South Asia [21]. Geological, anthropological, and industrial activities are the major sources of arsenic pollution in these areas [22]. In Bangladesh, arsenic contamination in the groundwater (e.g., tube well water) was first detected in 1993 and is now widespread in 50 out of 64 districts of the country [23]. Currently, arsenic is present at a very high level (>50 μg/L) in at least 30% of the tube wells used to provide drinking water and approximately 50 million people use arsenic-contaminated water for their daily activities [23,24]. One of the major contributors to arsenic mobilization in groundwater is phosphate (PO_4) rich fertilizer leaching [25]. Therefore, in addition to contaminated drinking water, Bangladeshis also face the risk of arsenic exposure through the consumption of fish raised in aquaculture ponds and rice irrigated by arsenic-polluted water [26,27]. Chronic exposure to arsenic causes serious health issues such as arsenicosis, type 2 diabetes, adverse pregnancy outcomes, and cardiovascular diseases [28–32]. In children, arsenic may cause lower respiratory diseases and diarrhea [33]. In a previous study in Bangladesh, it has been shown that the composition of gut microbiota was altered in children exposed to drinking water containing a high level of arsenic (>50 μg/L) compared to those exposed to drinking water containing low levels of arsenic [34]. The study also reported a higher abundance of antibiotic resistance and virulence genes in the gut microbiome of children that were exposed to higher arsenic levels. Based on this work, arsenic-exposed populations may face further risks of increased gut colonization with ARO due to arsenic-antibiotic co-resistance in enteric bacteria, but empirical evidence is lacking.

Resistance to arsenic might be ubiquitous among various microbial species. Arsenic-resistant bacteria have been isolated from various sources, including industrial wastewater [35], aquatic environments [36–38], and soil [39]. Arsenic resistance has also been reported among the members of the Enterobacteriaceae family [40,41]. Exposure to arsenic might play a role in the emergence and spread of antibiotic resistance among organisms in metal-contaminated environments [42]. One study has shown arsenic-induced antibiotic resistance in bacteria in laboratory conditions [15]. Bacteria may use similar mechanisms to become resistant to arsenic and antibiotics [43,44]. Therefore, it is likely that humans and animals that are exposed to arsenic may be colonized with ARO even without being exposed to antibiotics, but there is a lack of evidence to support this claim.

Given the public health significance of AMR, it is important to understand the drivers of AMR carriage, including the relationship between exposure to arsenic through drinking water and the fecal carriage of ARO. In this study, we apply a cross-sectional natural experiment type design to assess how arsenic exposure in drinking water correlates to antibiotic resistance in *E. coli* in drinking water samples and stool samples collected from households in Bangladesh. Specifically, we assess AMR in water and the feces of both mothers and children in two rural areas in which there is high (Hajiganj) and low (Matlab) levels of arsenic present in drinking water.

Results

Arsenic concentrations in household water samples

Household drinking water samples collected from the Hajiganj area had arsenic concentrations ranging from 223 μg/L to 729 μg/L with a median value of 481 μg/L (arsenic concentrations of individual households are listed in S1 Table). In contrast, arsenic concentrations in household water samples from the Matlab area ranged from 0 μg/L to 20 μg/L with a median

value of 0 μg/L. Therefore, the households enrolled in Hajiganj were exposed to high arsenic concentrations, and households in Matlab were exposed to low arsenic concentrations.

Prevalence and abundance of antibiotic-resistant *E. coli* in high and low arsenic areas

A total of 84% (251 of 300) samples across both sites, including 60 water samples, 100 stool samples from mothers (MS), and 91 stool samples from children (CS) were found to be positive for *E. coli*. Third-generation cephalosporin-resistant (3GCr) *E. coli* was found in a total of 67% (201 of 300) samples (24 water, 95 MS, and 82 CS), while 65% (194 of 300) samples (26 water, 98 MS, and 70 CS) were positive for fluoroquinolone-resistant (FQr) *E. coli*. A significantly higher proportion of water samples in Hajiganj were positive for 3GCr *E. coli*, FQr *E. coli*, or both compared to samples in Matlab (Chi-squared test, $p = 0.0001$; Table 1). Similarly, a significantly higher proportion of children in Hajiganj (94%) were colonized with 3GCr *E. coli*, FQr *E. coli*, or both than children in Matlab (76%) ($p = 0.0004$). However, no significant difference was found in the number of mothers colonized with 3GCr *E. coli* between Hajiganj and Matlab ($p = 0.5164$).

The abundance of total *E. coli*, 3GCr *E. coli*, and FQr *E. coli* in each sample was also determined. The mean number of total *E. coli* in water samples from Hajiganj (1.48 $\log_{10}$ CFU/100 ml; SD: 0.75) was significantly higher than that of Matlab (0.76 $\log_{10}$ CFU/100 ml; SD: 0.81) (Mann-Whitney U test, $p = 0.0003$; Fig 1A). However, no significant difference was found for the 3GCr and FQr *E. coli* in water samples between Hajiganj and Matlab. Likewise, no significant difference in the mean count of total *E. coli*, 3GCr *E. coli*, and FQr *E. coli* was found in MS and CS samples between the Hajiganj and Matlab areas (Fig 1B and 1C).

Antibiotic susceptibility of *E. coli* isolates

A total of 251 *E. coli* isolates (Hajiganj = 132, Matlab = 119) (one isolate per sample positive for *E. coli*) were tested for susceptibility against a panel of 16 antibiotics encompassing 11 antibiotic classes (S1 Fig). A total of 94% (235 of 251) of *E. coli* isolates across both sites [54% (126 of 235) from Hajiganj and 46% (109 of 235) from Matlab] were resistant to at least one class of antibiotic. Based on their susceptibility to different antibiotics, isolates were classified as multidrug-resistant (MDR), extended-spectrum β-lactamase (ESBL)-producing, 3GCr, and FQr. A significantly higher proportion of *E. coli* isolates from drinking water in Hajiganj were found to be MDR, ESBL-producing, 3GCr, and FQr compared to the isolates from Matlab (Chi-squared test, $p < 0.05$; Table 2). Similarly, a significantly higher proportion of MDR *E. coli*,

Table 1. Prevalence of total *E. coli* and antibiotic-resistant *E. coli* in water, mother stool (MS), and child stool (CS) in Hajiganj and Matlab.

E. coli	No. (%) of samples								
	Water			MS			CS		
	Hajiganj[1] ($n = 50$)	Matlab[2] ($n = 50$)	p-value	Hajiganj ($n = 50$)	Matlab ($n = 50$)	p-value	Hajiganj ($n = 50$)	Matlab ($n = 50$)	p-value
Total	34 (68)	26 (52)	0.0209	50 (100)	50 (100)	>0.99	48 (96)	43 (86)	0.0135
3GCr[3]	15 (30)	9 (18)	0.0469	48 (96)	47 (94)	0.5164	45 (90)	37 (74)	0.0032
FQr[4]	19 (38)	7 (14)	0.0001	50 (100)	48 (96)	0.0434	39 (78)	31 (62)	0.0136
3GCr/ FQr/both	24 (48)	11 (22)	0.0001	50 (100)	48 (96)	0.0434	47 (94)	38 (76)	0.0004

[1]Hajiganj, high arsenic exposure.

[2]Matlab, low arsenic exposure.

[3]3GCr, third-generation cephalosporin-resistant.

[4]FQr, fluoroquinolone-resistant.

https://doi.org/10.1371/journal.ppat.1010952.t001

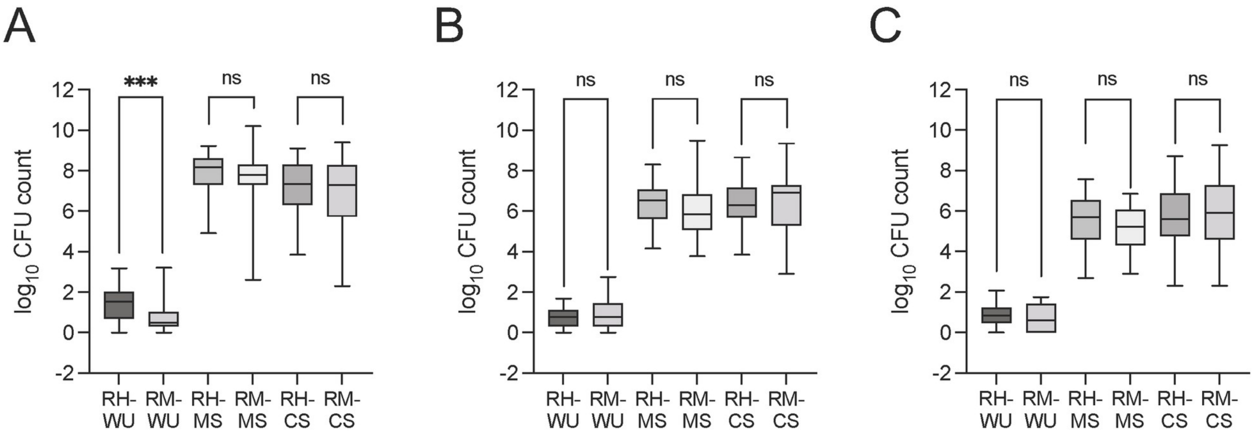

Fig 1. The abundance of total *E. coli* and antibiotic-resistant *E. coli* in water and stool samples from mothers and children in Hajiganj and Matlab. The number of A) total *E. coli*, B) third-generation cephalosporin-resistant *E. coli*, and C) fluoroquinolone-resistant *E. coli* in drinking water (WU), mother stool (MS), and child stool (CS) samples collected from the Hajiganj (RH, high arsenic exposure) and Matlab (RM, low arsenic exposure) areas were counted from the plates for each of the samples as described in the Methods section. The numbers were calculated as either CFU/100ml for water samples or CFU/g for MS and CS samples and transformed to $\log_{10}$ count. Each box represents the first and third quartile number of *E. coli* ($\log_{10}$ transformed) for one type of sample with the black bars representing the median count and error bars representing the minimum and maximum range of the counts, respectively. The asterisks indicate significance (***$p < 0.001$). 'ns' indicates not significant.

https://doi.org/10.1371/journal.ppat.1010952.g001

ESBL *E. coli*, and FQr *E. coli* isolates were found in CS samples in Hajiganj compared to Matlab ($p < 0.05$; Table 2). In contrast, there was no significant difference in the proportion of ESBL *E. coli*, 3GCr *E. coli*, and FQr *E. coli* in MS samples between Hajiganj and Matlab. A significantly higher proportion of *E. coli* isolates from water and CS samples in Hajiganj had a multiple antibiotic resistance (MAR) index of 0.2 or higher than isolates in Matlab ($p < 0.05$), suggesting that isolates in Hajiganj are frequently exposed to environments contaminated with antimicrobial agents (Table 2).

Table 2. Antibiotic susceptibility of *E. coli* isolates obtained from water, mother stool (MS), and child stool (CS) in Hajiganj and Matlab.

Antibiotic resistance pattern	*No. (%) of *E. coli* isolates from								
	Water			MS			CS		
	Hajiganj[1] (n = 34)	Matlab[2] (n = 26)	p-value	Hajiganj (n = 50)	Matlab (n = 50)	p-value	Hajiganj (n = 48)	Matlab (n = 43)	p-value
MDR[3]	21 (62)	12 (46)	0.0232	43 (86)	36 (72)	0.0151	46 (96)	37 (86)	0.0135
ESBL[4]	17 (50)	9 (35)	0.0319	44 (88)	44 (88)	>0.9999	43 (89)	32 (74)	0.0063
3GCr[5]	17 (50)	9 (35)	0.0319	48 (96)	47 (94)	0.5164	46 (96)	40 (93)	0.3521
FQr[6]	14 (41)	5 (19)	0.0007	14 (28)	9 (18)	0.0929	23 (48)	13 (30)	0.0091
MAR[7]	25 (73)	12 (46)	0.0001	45 (90)	42 (84)	0.2071	48 (48)	39 (43)	0.0021

[1]Hajiganj, high arsenic exposure.

[2]Matlab, low arsenic exposure.

[3]MDR, multidrug-resistant.

[4]ESBL, extended-spectrum β-lactamase-producing.

[5]3GCr, third-generation cephalosporin-resistant.

[6]FQr, fluoroquinolone-resistant.

[7]MAR, multiple antibiotic resistance (MAR) index of 0.2 or higher.

*The number represents one *E. coli* isolate per sample. *E. coli* isolates were obtained from antibiotic (cefotaxime/ciprofloxacin) supplemented plates. For samples showing no growth on antibiotic plates, *E. coli* isolates were obtained from antibiotic-free plates.

https://doi.org/10.1371/journal.ppat.1010952.t002

Table 3. Prevalence of ESBL and diarrheagenic genes among *E. coli* isolates obtained from water, mother stool (MS), and child stool (CS) in Hajiganj and Matlab.

Genes	No. (%) of *E. coli* isolates							
	Water		MS		CS		Total	
	Hajiganj[1] (n = 34)	Matlab[2] (n = 26)	Hajiganj (n = 50)	Matlab (n = 50)	Hajiganj (n = 48)	Matlab (n = 43)	Hajiganj (n = 132)	Matlab (n = 119)
ESBL gene								
$bla_{CTX\text{-}M\text{-}1}$	15 (44.1)	9 (34.6)	40 (80)	44 (88)	41 (85.4)	35 (81.4)	96 (72.7)	88 (73.9)
bla_{TEM}	8 (23.5)	4 (15.4)	13 (26)	10 (20)	21 (43.7)	13 (30.2) *	42 (31.8)	27 (22.7)
$bla_{OXA\text{-}1}$	2 (5.9)	1 (3.8)	2 (4)	3 (6)	8 (16.7)	3 (7) *	12 (9.11)	7 (5.9)
bla_{SHV}	1 (2.9)	0 (0)	0 (0)	0 (0)	0 (0)	0 (0)	1 (0.8)	0 (0)
Diarrheagenic gene[3]								
aaiC	2 (5.9)	1 (3.8)	5 (10)	0 (0) *	3 (6.2)	4 (9.3)	10 (7.6)	5 (4.2)
aatA	2 (5.9)	3 (11.5)	4 (8)	3 (6)	6 (12.5)	9 (20.9)	12 (9.1)	15 (12.6)
bfpA	0 (0)	1 (3.8)	2 (4)	2 (4)	2 (4.2)	2 (4.6)	4 (3)	5 (4.2)
eaeA	0 (0)	1 (3.8)	2 (4)	2 (4)	2 (4.2)	1 (2.3)	4 (3)	4 (3.4)

[1]Hajiganj, high arsenic exposure.

[2]Matlab, low arsenic exposure.

[3] EAEC (*aaiC*, *aatA*), EPEC (*bfpA*, *eaeA*), EIEC (*ipaH*, *ial*), ETEC (*lt*, *st*), STEC (*stx1*, *stx2*); None of the isolates were positive for *ipaH*, *ial*, *lt*, *st*, *stx1*, and *stx2* genes.

* indicates statistical significance (p <0.05) for the number of isolates positive in Hajiganj compared to Matlab.

https://doi.org/10.1371/journal.ppat.1010952.t003

Prevalence of ESBL and diarrheagenic genes in *E. coli* from high and low arsenic areas

Of the four ESBL genes tested among 251 *E. coli* isolates from both sites, $bla_{CTX\text{-}M\text{-}1}$ was identified as the most prevalent (73%, n = 184) gene followed by bla_{TEM} (27%, n = 69), $bla_{OXA\text{-}1}$ (8%, n = 19) and bla_{SHV} (0.4%, n = 1). Overall, 74% (98 of 132) isolates in Hajiganj and 75% (89 of 119) isolates in Matlab were positive for at least one ESBL gene. Among diarrheagenic genes, enteroaggregative *E. coli* (EAEC)-specific *aatA* and *aaiC* genes were present in 11% (27 of 251) and 6% (15 of 251) of all isolates, respectively. Enteropathogenic *E. coli* (EPEC)-specific *bfpA* and *eaeA* genes were present in 4% (9 of 251) and 3% (8 of 251) of all isolates, respectively. None of the isolates were positive for enteroinvasive *E. coli* (EIEC) (*ipaH*, *ial*), enterotoxigenic *E. coli* (ETEC) (*lt*, *st*), or Shiga toxin-producing *E. coli* (STEC) (*stx1*, *stx2*) specific genes. Overall, 18% (24 of 132) of isolates in Hajiganj and 21% (25 of 119) of isolates in Matlab tested positive for *E. coli* diarrheagenic genes. The prevalence of ESBL and diarrheagenic genes in each group of *E. coli* isolates from both Hajiganj and Matlab areas are presented in Table 3.

Arsenic resistance among *E. coli* isolates

About 80% (200 of 251) of all *E. coli* isolates including 82% (108 of 132) of isolates from Hajiganj and 77% (92 of 119) of isolates from Matlab were identified as arsenic-resistant based on the minimum inhibitory concentration (MIC) of ≥5 mM of sodium arsenite [As(III)]. A significantly higher proportion of isolates from water samples in Hajiganj (79%, 27 of 34) were found to be resistant to arsenic compared to the isolates from water samples in Matlab (54%, 14 of 26) (Chi-squared test, p = 0.0002; Table 4). However, there was no significant difference in the prevalence of arsenic-resistant *E. coli* in MS or CS samples between the Hajiganj and Matlab areas (Table 4).

The presence of characteristic arsenic resistance genes including *arsA*, *arsB*, *arsC*, *arsR*, and plasmid-specific *arsR* and *arsD* was tested by PCR assays. None of the *E. coli* isolates tested positive for the *arsA* gene. The *arsB* gene was present in 52% (131 of 251) of *E. coli* isolates from both sites, including in 67% (89 of 132) of isolates from Hajiganj and in 35% (42 of 119)

Table 4. The minimum inhibitory concentration (MIC) of *E. coli* isolates against sodium arsenite [(As(III)].

Type of samples (no. of isolates)	No. (%) of arsenic-resistant isolates	As(III) MIC (mM)[1]		
		MIC Range[2]	MIC50[3]	MIC90[4]
Hajiganj				
Water (n = 34)	27 (79.4) *	4–8	5.5	7.0
Mother stool (n = 50)	43 (86) [ns]	3–10	5.5	7.0
Child stool (n = 48)	38 (79.2) [ns]	0.5–10	5.5	6.5
Matlab				
Water (n = 26)	14 (53.8)	3–8.5	4.5	7.0
Mother stool (n = 50)	42 (84)	0.5–7.5	5.5	7.0
Child stool (n = 43)	36 (83.7)	0.5–7.5	5.5	7.5

[1]MIC of As(III) was determined by micro broth dilution method as described in the Methods section.

[2]Range of MIC (mM) of all isolates tested.

[3]MIC50 indicates the minimum inhibitory concentration required to inhibit the growth of 50% of bacterial isolates.

[4]MIC90 indicates the minimum inhibitory concentration required to inhibit the growth of 90% of bacterial isolates.

* indicates statistical significance (p <0.05) for the number of isolates resistant to arsenic in Hajiganj compared to Matlab, 'ns' indicates non-significant.

https://doi.org/10.1371/journal.ppat.1010952.t004

of isolates from Matlab. A significantly higher proportion of *E. coli* isolates from water, MS, and CS samples from Hajiganj were positive for *arsB* compared to the corresponding group of isolates from Matlab (Chi-squared test, p <0.01; Fig 2A). In the case of FQr and 3GCr *E. coli*, water, MS, and CS isolates from Hajiganj were predominantly positive for *arsB* compared to the isolates from Matlab (p <0.01; Fig 2B and 2C). The *arsC* gene was present in 90% (225 of 251) of all *E. coli* isolates (Hajiganj = 124, Matlab = 101), while *arsR* was present in 95% (238 of 251) of all *E. coli* isolates (Hajiganj = 125, Matlab = 113). Plasmid-specific *arsR* and *arsD* were present in 23 (Hajiganj = 11, Matlab = 12) and 34 (Hajiganj = 7, Matlab = 27) *E. coli* isolates, respectively. Overall, 17% (43 of 251) of all *E. coli* isolates carried either plasmid-specific *arsR*, *arsD*, or both genes.

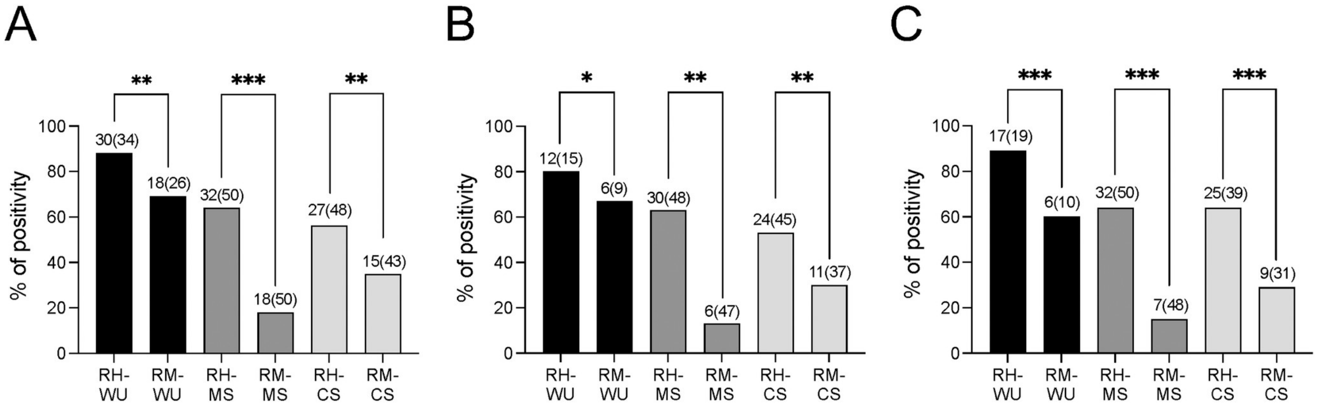

Fig 2. Prevalence of *arsB* gene among *E. coli* isolates. Prevalence of *E. coli* isolates positive for *arsB* gene in A) total no. of *E. coli* isolates, B) third-generation cephalosporin-resistant *E. coli* isolates, and C) fluoroquinolone-resistant *E. coli* isolates obtained from water (WU), mother stool (MS), and child stool (CS) samples collected from the Hajiganj (RH, high arsenic exposure) and Matlab (RM, low arsenic exposure) areas in Bangladesh. Each bar represents the percentage of *E. coli* isolates that are positive for the *arsB* gene. The numbers on top of each bar indicate the number of *E. coli* isolates that are positive for the *arsB* gene and the numbers in parenthesis indicate the total number of *E. coli* isolates for each type of sample. The asterisks indicate significance (*p <0.05, **p <0.01, ***p <0.001).

https://doi.org/10.1371/journal.ppat.1010952.g002

Correlation between arsenic resistance and antibiotic resistance among 3GCr *E. coli* isolates

Co-resistance to arsenic and antibiotic classes including penicillin, cephalosporin, tetracycline, fluoroquinolone, macrolide, and trimethoprim-sulfamethoxazole was found in a significantly higher proportion of *E. coli* isolates from water samples in Hajiganj compared to Matlab (Chi-squared test, $p < 0.05$; Fig 3A). Similarly, a significantly higher proportion of *E. coli* isolates from MS samples in Hajiganj had co-resistance to arsenic and antibiotic classes such as penicillin, cephalosporin, tetracycline, and macrolide than *E. coli* isolates from MS in Matlab ($p < 0.05$; Fig 3B). In the case of CS samples, co-resistance to arsenic and antibiotic classes (penicillin, cephalosporin, fluoroquinolone, and macrolide) was found in a significantly higher proportion of *E. coli* isolates from Hajiganj than Matlab ($p < 0.05$; Fig 3C).

Next, the association between arsenic and antibiotic resistance was observed among all *E. coli* isolates from both areas. The odds of arsenic-resistant *E. coli* to be resistant to β-lactam antibiotics including ampicillin (OR: 3.4, 95% CI: 1.4, 8.1, $p < 0.01$), cefotaxime (OR: 2.5, 95% CI: 1.2, 5.3, $p < 0.05$), and ceftriaxone (OR: 3.4, 95% CI: 1.6, 7.0, $p < 0.01$) were higher compared to that of arsenic-sensitive *E. coli* isolates (Fig 4). In contrast, arsenic-resistant isolates were less likely to be resistant to tetracycline (OR: 0.4, 95% CI: 0.2, 0.8, $p < 0.05$), trimethoprim-sulfamethoxazole (OR: 0.4, 95% CI: 0.2, 0.8, $p < 0.01$) and ciprofloxacin (OR: 0.4, 95% CI: 0.2, 0.8, $p < 0.05$) compared to that of arsenic-sensitive isolates.

Whole genome sequence analysis of *E. coli* isolates

Whole genome sequence (WGS) analysis of 30 representative isolates from Hajiganj ($n = 15$) and Matlab ($n = 15$) showed differential characteristics. *E. coli* phylogroup analysis showed that among Hajiganj isolates, phylogroup A was the predominant ($n = 7$) followed by phylogroup B1 ($n = 3$), phylogroup C ($n = 2$), phylogroup B2 ($n = 1$), phylogroup E ($n = 1$), and an unidentified phylogroup ($n = 1$). For Matlab isolates, phylogroup D was the most common ($n = 7$), followed by phylogroup A ($n = 3$), phylogroup B1 ($n = 3$), and phylogroup B2 ($n = 2$) (Fig 5).

Grouping of isolates based on whole genome multilocus sequence typing (wgMLST) revealed a heterogeneous distribution of sequence types (STs) among isolates from both areas. A total of 18 STs were identified among the 30 isolates and there was less overlap of STs between isolates from the Hajiganj and Matlab areas. Isolates from Hajiganj belonged to STs 10, 131, 224, 226, 315, 410, 602, 3018, and 3744, while isolates from Matlab were grouped into STs 38, 70, 131, 394, 443, 1290, 2705, 2280, 3580, and 8131. One MS isolate from Matlab was not identified as any known ST type, therefore it is mentioned as a novel ST.

Analysis of the arsenic resistance gene operon among isolates revealed that complete *arsRBC* operon was present in 80% (24 of 30) of the isolates. Six isolates had incomplete *arsRBC* operon; among these, three isolates (one isolate from Hajiganj and two from Matlab) did not have the *arsR* and *arsB* genes and three other isolates from Matlab had a truncated *arsR* gene (Fig 5). Three isolates that were negative for *arsR* and *arsB* genes also had the lowest MIC against As(III). All three isolates belonged to the phylogroup B2 and ST131.

The resistome analysis showed that both Hajiganj and Matlab isolates shared antibiotic resistance genes (Fig 6A). However, isolates from Hajiganj were distinctively positive for a few antibiotic resistance genes that were not present in isolates from Matlab areas. These include $bla_{\text{TEM-128}}$ and $bla_{\text{TEM-135}}$ for β-lactam, *qnrS4* for fluoroquinolone, *sul3* for sulfonamide, *aph (3')-Ia* for aminoglycoside, *cmlA1* for phenicol, and *tet(b)* and *tet(D)* for tetracycline. The ESBL *E. coli* isolates from both Hajiganj and Matlab were positive for a catalog of virulence genes that are broadly categorized as adhesins, toxins, iron acquisition, and regulators. Most of

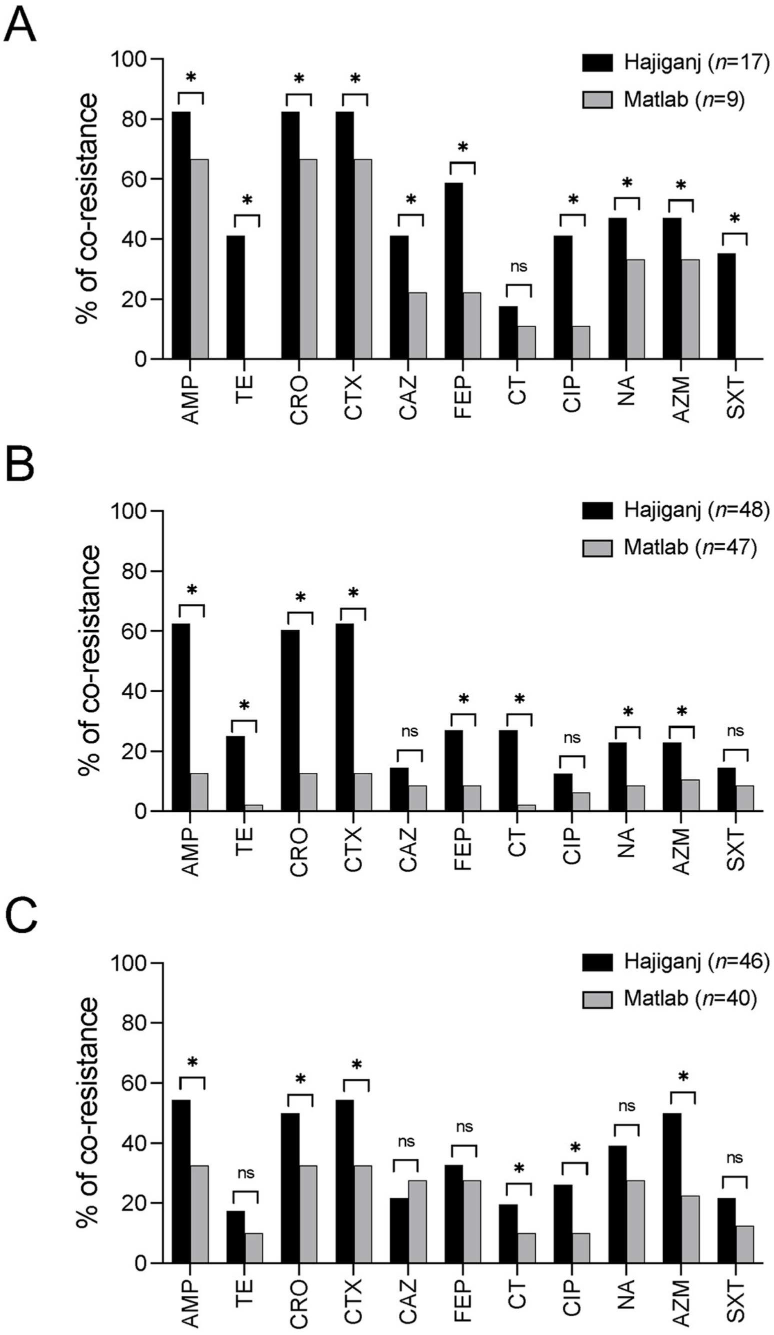

Fig 3. Co-occurrence of arsenic and antibiotic resistance in extended-spectrum β-lactamase (ESBL)-producing *E. coli* isolates. Co-resistance to arsenic and antibiotic resistance of ESBL *E. coli* isolates in A) drinking water, B) mother stool, and C) child stool samples in Hajiganj (high arsenic exposure) and Matlab (low arsenic exposure) was determined by selecting bacterial isolates that showed resistance to any of the third-generation cephalosporin antibiotics (CRO, ceftriaxone, CTX, cefotaxime; CAZ, ceftazidime; FEP, cefepime) and were *arsB* gene positive by PCR assays. '*n*' indicates the number of third-generation cephalosporin-resistant bacterial isolates for each group of samples. AMP, ampicillin; TE, tetracycline; CRO, ceftriaxone, CTX, cefotaxime; CAZ, ceftazidime; FEP, cefepime; CT, colistin; CIP, ciprofloxacin; NA, nalidixic acid; AZM, azithromycin; SXT, trimethoprim-sulfamethoxazole. The asterisks indicate significance (*p <0.05).

https://doi.org/10.1371/journal.ppat.1010952.g003

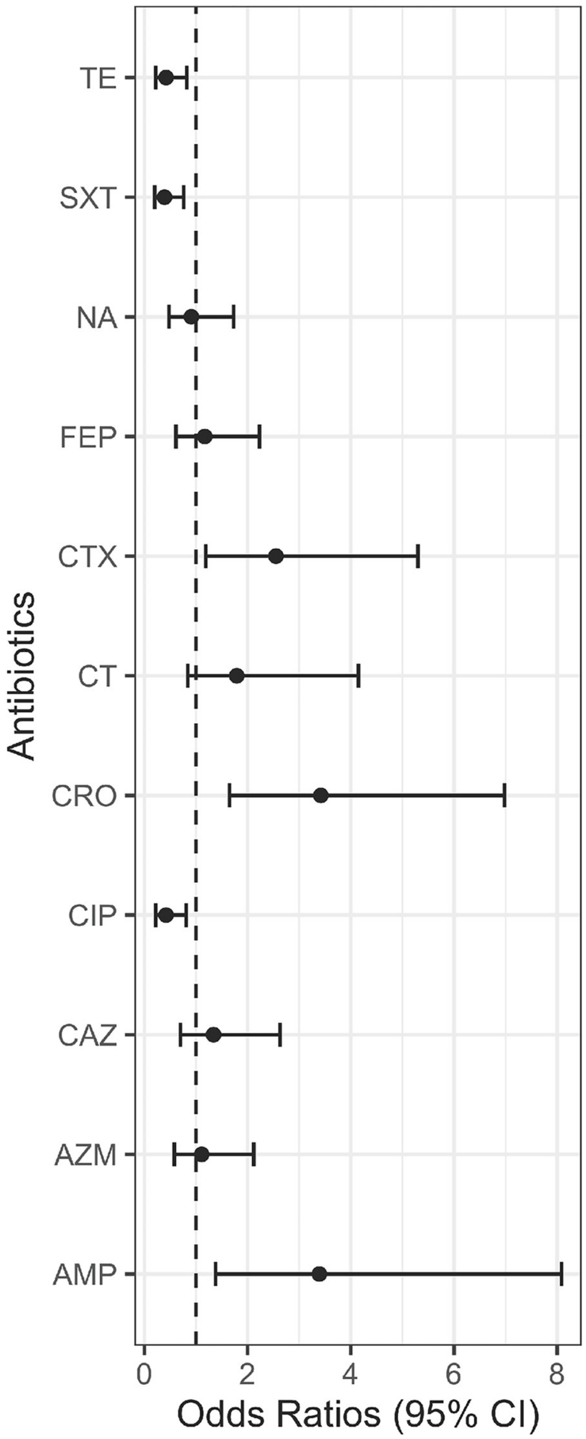

Fig 4. Forest plot for the odds ratios (OR) along with their 95% confidence intervals (CIs). The black dot indicates the OR of being resistant to the corresponding antibiotics in the arsenic-resistant group of isolates compared to the arsenic-sensitive group of isolates. The whiskers are the spread of the CIs of the OR. The dotted line indicates the OR of 1 which implies no association. AMP, ampicillin; TE, tetracycline; CRO, ceftriaxone, CTX, cefotaxime; CAZ, ceftazidime; FEP, cefepime; CT, colistin; CIP, ciprofloxacin; NA, nalidixic acid; AZM, azithromycin; SXT, trimethoprim-sulfamethoxazole.

https://doi.org/10.1371/journal.ppat.1010952.g004

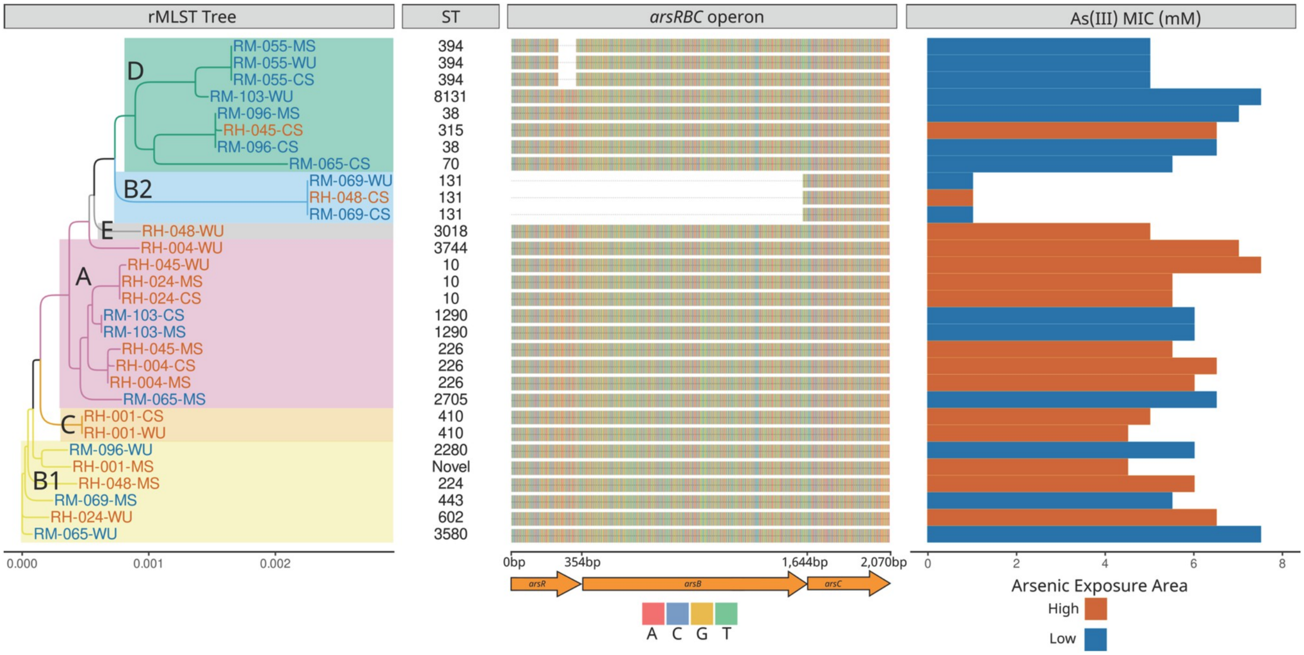

Fig 5. Neighbor-joining tree based on ribosomal MLST (rMLST) allele sequences for 30 *E. coli* isolates. Sequence types (STs), *ars* operon, and minimum inhibitory concentration (MIC) to arsenite are shown next to the tree. Colored leaves and shaded regions indicate the phylogroups. RH and RM in isolate ID indicate if the isolate was obtained from Hajiganj (high arsenic exposure) or Matlab (low arsenic exposure) areas, respectively. WU, MS, and CS in isolate ID indicate isolates from drinking water, mother stool, and child stool, respectively.

https://doi.org/10.1371/journal.ppat.1010952.g005

the virulence genes were commonly distributed between isolates from low and high arsenic areas resulting in no clustering of isolates (Fig 6B). However, several genes including *papA*, *agg3D*, *agg3A*, *agg3C*, *agg3B*, and *agg5A* were present in the Matlab isolates, while absent in Hajiganj isolates. Overall, isolates from the Matlab areas were positive for a higher number of virulence genes compared to the isolates from the Hajiganj areas, although the difference was not statistically significant.

Factors associated with co-resistance to arsenic and antibiotics among *E. coli* isolates

Fecal carriage of 3GCr and FQr *E. coli* among children in the Hajiganj and Matlab areas was not associated with the children's demographic characteristics including sex, maternal use of antibiotics, and mode of delivery (Table 5). Similarly, no significant association was observed in the fecal carriage rate of 3GCr and FQr *E. coli* among the mothers with respect to their age, previous antibiotic exposure, and diarrheal incidence.

Discussion

In this observational study, we found that fecal carriage of antibiotic-resistant *E. coli* amongst children, but not amongst mothers, was associated with exposure to arsenic via contaminated drinking water. Third-generation cephalosporin (3GC) and fluoroquinolones (FQs) are the two most commonly prescribed/used antibiotic classes for the treatment of bacterial infections in the hospital and community settings in Bangladesh [45,46]. The high rate (94%) of colonization of children with ARO (3GCr/FQr/both) in the arsenic-contaminated area is alarming given the increased risks of adverse health outcomes from ARO carriage in children. Specifically, the gut microbiome during childhood has a long-lasting effect on overall health in

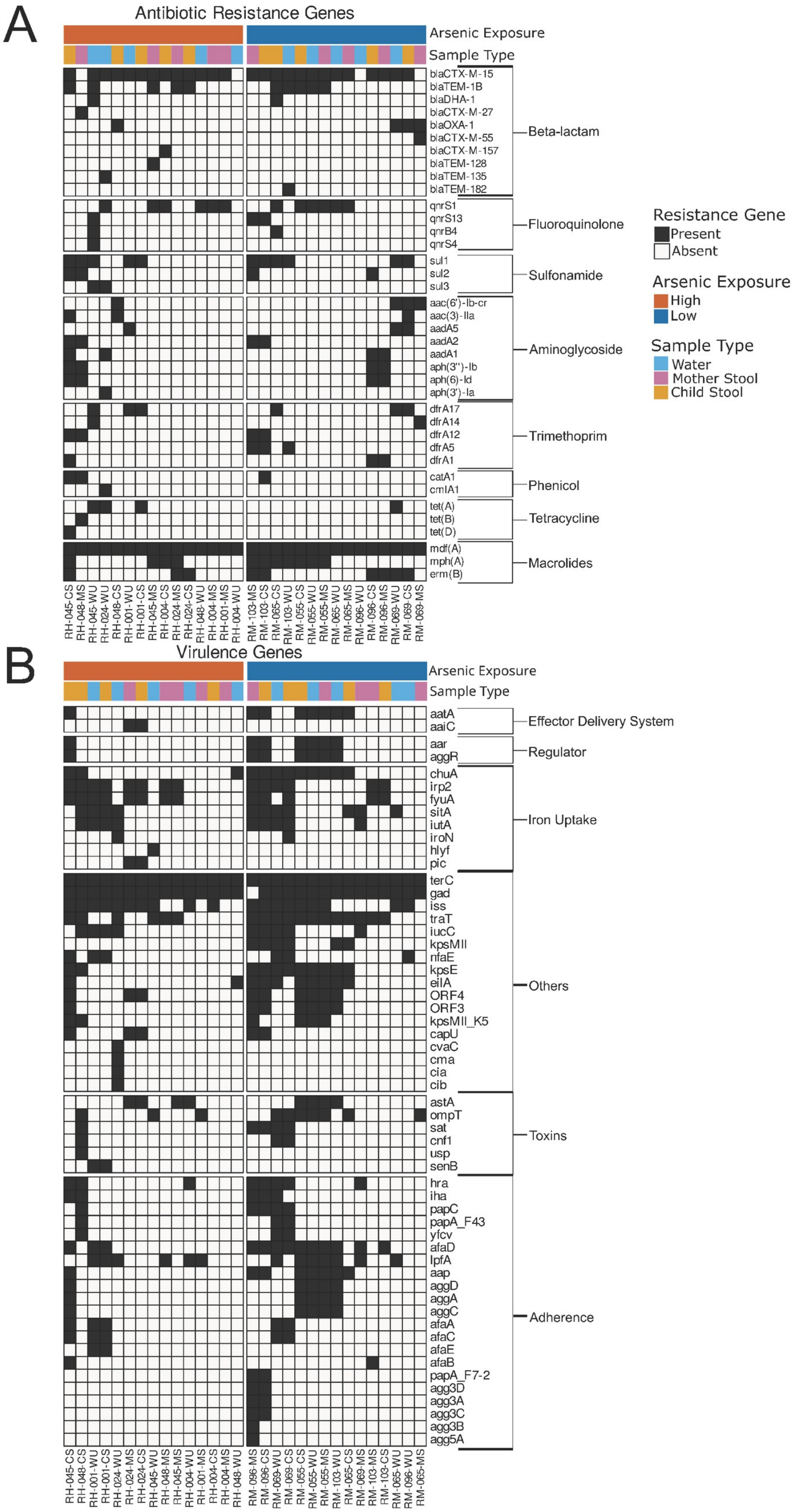
A
Antibiotic Resistance Genes
Arsenic Exposure
Sample Type
blaCTX-M-15
blaTEM-1B
blaDHA-1
blaCTX-M-27
blaOXA-1
blaCTX-M-55
blaCTX-M-157
blaTEM-128
blaTEM-135
blaTEM-182
Beta-lactam
qnrS1
qnrS13
qnrB4
qnrS4
Fluoroquinolone
sul1
sul2
sul3
Sulfonamide
aac(6')-Ib-cr
aac(3)-IIa
aadA5
aadA2
aadA1
aph(3'')-Ib
aph(6)-Id
aph(3')-Ia
Aminoglycoside
dfrA17
dfrA14
dfrA12
dfrA5
dfrA1
Trimethoprim
catA1
cmlA1
Phenicol
tet(A)
tet(B)
tet(D)
Tetracycline
mdf(A)
mph(A)
erm(B)
Macrolides
RH-045-CS
RH-048-MS
RH-045-WU
RH-024-WU
RH-048-CS
RH-001-WU
RH-001-CS
RH-045-MS
RH-004-CS
RH-024-MS
RH-024-CS
RH-048-WU
RH-004-MS
RH-001-MS
RH-004-WU
RM-103-MS
RM-103-CS
RM-065-CS
RM-103-WU
RM-055-CS
RM-055-WU
RM-055-MS
RM-065-WU
RM-065-MS
RM-096-WU
RM-096-CS
RM-096-MS
RM-069-WU
RM-069-CS
RM-069-MS
Resistance Gene
Present
Absent
Arsenic Exposure
High
Low
Sample Type
Water
Mother Stool
Child Stool
B
Virulence Genes
Arsenic Exposure
Sample Type
aatA
aaiC
Effector Delivery System
aar
aggR
Regulator
chuA
irp2
fyuA
sitA
iutA
iroN
hlyf
pic
Iron Uptake
terC
gad
iss
traT
iucC
kpsMII
nfaE
kpsE
eilA
ORF4
ORF3
kpsMII_K5
capU
cvaC
cma
cia
cib
Others
astA
ompT
sat
cnf1
usp
senB
Toxins
hra
iha
papC
papA_F43
yfcv
afaD
lpfA
aap
aggD
aggA
aggC
afaA
afaC
afaE
afaB
papA_F7-2
agg3D
agg3A
agg3C
agg3B
agg5A
Adherence
RH-045-CS
RH-048-CS
RH-001-WU
RH-001-CS
RH-024-WU
RH-024-MS
RH-024-CS
RH-045-WU
RH-048-MS
RH-045-MS
RH-004-WU
RH-001-MS
RH-004-CS
RH-004-MS
RH-048-WU
RM-096-MS
RM-096-CS
RM-069-WU
RM-069-CS
RM-055-CS
RM-055-WU
RM-055-MS
RM-103-WU
RM-065-CS
RM-069-MS
RM-103-MS
RM-103-CS
RM-065-WU
RM-096-WU
RM-065-MS

Fig 6. Heatmaps of resistome and virulence genes of *E. coli* isolates. The distribution of A) antibiotic resistance genes, and B) virulence genes retrieved from whole genome sequencing data of 30 *E. coli* isolated from water, mother stool, and child stool samples from the Hajiganj (high arsenic exposure) and Matlab (low arsenic exposure) areas. The heatmap shows genes' presence (black box) or absence (white box). Gene names are listed on the right. *E. coli* isolate IDs are listed at the bottom. RH and RM in isolate ID indicate if the isolate was obtained from the Hajiganj and Matlab areas, respectively. WU, MS, and CS in isolate ID indicate isolates from drinking water, mother stool, and child stool, respectively.

https://doi.org/10.1371/journal.ppat.1010952.g006

adulthood. Moreover, gut colonization with ARO increases the risk of drug-resistant infections, including urinary tract and bloodstream infections and neonatal meningitis [47–49]. Additionally, gut colonization with ARO represents a gene pool for antimicrobial resistance genes that can be exchanged with other human pathogens in the gut.

Table 5. Demographic characteristics of children and mothers colonized with third-generation cephalosporin-resistant (3GCr) or fluoroquinolone-resistant (FQr) *E. coli* in areas with and without arsenic exposure through drinking water.

Characteristics	No. (%) of samples positive for 3GCr *E. coli**		No. (%) of samples positive for FQr *E. coli**	
	Hajiganj[1] (n = 50)	Matlab[2] (n = 50)	Hajiganj (n = 50)	Matlab (n = 50)
Children				
Sex				
Male	25/27 (93)	19/28 (68)	21/27 (78)	14/28 (50)
Female	20/23 (87)	18/22 (82)	18/23 (78)	17/22 (77)
Age				
≤6 months	22/25 (88)	11/19 (58)	21/27 (78)	14/28 (50)
>6 months	23/25 (92)	26/31 (84)	18/23 (78)	17/22 (77)
Mode of delivery[3]				
CS	17/18 (94)	10/14 (71)	14/18 (78)	8/14 (64)
NVD	15/18 (83)	12/14 (86)	14/18 (78)	9/14 (64)
Previous antibiotic consumption				
Yes	30/33 (91)	23/30 (77)	27/33 (82)	19/30 (82)
No	15/17 (88)	14/19 (74)	12/17 (71)	12/19 (63)
Diarrhea				
Yes	6/7 (86)	3/3 (100)	4/7 (57)	2/3 (67)
No	39/43 (91)	31/42 (74)	35/43 (81)	27/42 (64)
Mothers				
Age				
<25 years	28/29 (97)	21/22 (95)	29/29 (100)	22/22 (100)
≥25 years	19/20 (95)	26/27 (96)	20/20 (100)	26/27 (96)
Previous antibiotic consumption				
Yes	7/7 (100)	7/7 (100)	7/7 (100)	7/7 (100)
No	41/43 (95)	40/43 (93)	43/43 (100)	41/43 (95)
Diarrhea				
Yes	5/5 (100)	1/1 (100)	5/5 (100)	1/1 (100)
No	43/45 (96)	44/47 (94)	45/45 (100)	45/47 (96)

[1]Hajiganj, high arsenic exposure.

[2]Matlab, low arsenic exposure.

[3]Information about the mode of delivery was available for 36 children in the high arsenic area and 24 children in the low arsenic area.

*None of the p-values are statistically significant after adjusting for multiple comparisons using the Holm-Bonferroni test.

CS: Caesarian section; NVD: Normal vaginal delivery.

https://doi.org/10.1371/journal.ppat.1010952.t005

The mothers in both regions (Hajiganj and Matlab) had high rates of fecal ARO carriage. Households in Matlab are exposed to a low level of arsenic through drinking water because of the installation of deep tube wells, which were installed in the area during 2001–2006 [50]. Before 2001, arsenic contamination in wells in Matlab was likely similar to those observed now in Hajiganj. The average age of mothers enrolled in this study was 25 years, so they were likely exposed to high arsenic levels during their childhood, which might have a role in shaping their gut microbiomes including a higher carriage of ARO. Besides, diet could be an important driver for chronic exposure to arsenic among the adult population in the area. Although mothers from Matlab households do not consume arsenic-contaminated water, they might be exposed to arsenic via the food chain, especially through the staple food rice and leafy vegetables which are irrigated with arsenic-contaminated water generally extracted from shallow groundwater aquifers. It has been reported that the relative contribution of dietary arsenic sources becomes more important when there is a lower risk of arsenic exposure via drinking water [51]. A previous study in Bangladesh reported that rice and leafy vegetables can absorb the highest amounts of arsenic depending on the cooking practices [52]. In our study, we did not focus on the dietary habits or food preparation practices among the households, and we also did not analyze the food samples for arsenic. Therefore, further studies are required to identify the other sources of arsenic exposure in areas where arsenic-free drinking water is accessible to the community.

A higher proportion of *E. coli* isolates from drinking water samples in Hajiganj were resistant to different classes of antibiotics than isolates from water samples in Matlab. Bacteria exposed to arsenic-contaminated groundwater may induce or select bacterial adaptations to one or more antibiotics through co-selection of resistance. This is in agreement with previous studies that organisms isolated from extreme environmental conditions such as industrial or agricultural wastewater have shown arsenic-mediated antibiotic resistance *in vitro* [15,42,53]. In a related study, 6 h arsenic exposure [As(III) of 0.2–1 mg/L] in drinking water increased bacterial resistance to cephalosporin, tetracycline, and erythromycin as well as increased the relative abundance of antibiotic resistance genes in a bacterial community [42]. In this study, we found that the co-resistance to arsenic and antibiotics was more prevalent among 3GCr *E. coli* in high arsenic areas compared to low arsenic areas. In particular, arsenic-resistant isolates were more likely to be resistant to β-lactam antibiotics including 3GC suggesting that the use of these antibiotics needs to be evaluated carefully in areas with high arsenic contamination. We also found that arsenic-resistant bacteria were less likely to be resistant to fluoroquinolone, chloramphenicol, and tetracycline antibiotics. Notably, the finding about tetracycline stands in contrast to previous *in vitro* studies showing bacterial exposure to arsenic promotes bacterial resistance to the tetracycline [15,42]. The difference in our study may be that tetracycline resistance prevalence is very high in both study areas, resulting in insufficient variability between sites to observe a significant difference.

This study finds that antibiotic-resistant *E. coli* are also resistant to arsenic and carry arsenic resistance genes. This imposes a greater public health risk because these bacteria can survive in water with high arsenic concentrations, which increases antibiotic resistance through selection for co-resistance and promotes antibiotic resistance gene transmission to other organisms via horizontal gene transmission. Notably, none of the isolates in our study carry *arsA*, a required gene when resistance to arsenic is due to *arsRDABC* operon. This indicates the presence of a simpler *arsRBC* operon in these isolates, which is also supported by a previous study [34]. Isolates that possess *arsRDABC* operon are more resistant to arsenic than those expressing *arsRBC* operon [54]. The absence of *arsB* in some isolates corresponds to the loss of arsenic resistance. From the WGS analysis, three isolates (one from Hajiganj and two from Matlab) had incomplete *ars* operon (missing *arsR* and *arsB*) and were sensitive to arsenic. Interestingly,

all three arsenic-sensitive isolates belong to ST131 and phylogroup B2. This finding is similar to a previous report by Sutterlin *et al* where they found that the majority of arsenic-sensitive *E. coli* isolates belonged to phylogroup B2 [40]. Plasmid-specific *arsR* and *arsD* genes were present in some isolates, indicating that some of the isolates carry these genes in the plasmids, as has been previously shown [55,56]. Colocation of arsenic and antibiotic resistance on plasmids among isolates in this study may explain the presence of dual resistance and would provide a mechanism for horizontal gene transfer of multiple resistances to other bacteria.

Analysis of isolates for *E. coli* diarrheagenic genes revealed that only a few isolates possessed genes related to EPEC and EAEC. Further, these organisms were not phylogenetically clustered based on location (high versus low arsenic exposure). However, from the WGS analysis of 30 *E. coli* isolates, we observed a higher number of virulence genes in isolates from the low arsenic area than in the high arsenic area. While the virulence mechanism is different from the resistance mechanism for both antibiotics and heavy metals, this difference in diversity may be due to the fitness cost of the isolates in high arsenic areas. Further studies are needed to understand the impact of heavy metal exposure on bacterial virulence properties. Several studies have reported a higher incidence of diarrheal diseases among children in arsenic-endemic areas [33,57–59]. However, whether it is due to the higher prevalence of diarrheal pathogens in the area or due to the vulnerability of the population to diarrhea with respect to their arsenic exposures, hygiene, sanitation, or access to clean drinking water supply is not well explained.

A primary limitation of this study is that the presence of arsenic in groundwater is spatially correlated, so the study participants with and without arsenic exposure could not be randomly chosen. The selection of households focused on two neighboring upazillas, which despite similar lifestyles and food habits, are still distinct neighborhoods, and as such there may be neighborhood-level differences beyond arsenic in water that contribute to the observed differences. Additionally, the fecal carriage of AMR may be driven by other sources of arsenic, including the food chain. Future studies may consider measuring the concentration of arsenic in urine samples of mothers and children from selected households before sampling to determine if arsenic concentrations in water are related to differences in arsenic burden. In addition, analyses were based on one isolate from each sample to compare the characteristics of organisms between the two communities, which does not necessarily fully represent the bacterial community diversity in samples. However, given the findings from this observational study, further studies can be pursued on the impact of arsenic in drinking water on antibiotic resistance carriage, such as randomized controlled trials potentially involving a stepped wedge intervention design focused on reducing arsenic in drinking water.

Our study suggests a plausible link between arsenic exposure and carriage of ARO among children in the study communities, which is an emerging dimension of the arsenic problem. This issue further complicates the existing risks to public health due to both arsenic exposure and AMR infections. Developing a sustainable solution to reduce arsenic exposures in Bangladesh would not only counter the associated health risks due to arsenic toxicity but may also reduce the burden of AMR in the community. Overall, this study highlights the urgent need to reduce arsenic exposures in these communities.

Methods

Ethics statement

The study (PR-16086) involving human participants was reviewed and approved by the research and the ethical review committees of the International Centre for Diarrhoeal Disease Research, Bangladesh (icddr,b). The review committees also monitored the progress of this

study. Written informed consent to participate in this study was provided by the mothers themselves and for their children by the participants' legal guardians.

Study design, site selection, and sample collection

Two rural upazillas (sub-districts)—(Hajiganj and Matlab)—in the Chandpur district of Bangladesh were selected for this study. Hajiganj has a history of high arsenic concentrations in drinking water, while the arsenic concentrations in drinking water in Matlab are generally low. These two populations presented the opportunity to study the effect of arsenic exposure on gut colonization with AMR bacteria. A total of 100 households that included 50 households in each upazilla were conveniently selected based on the following criteria, 1) having children of <1 year of age, and 2) members are exposed to high (>100 μg/L) versus low (<20 μg/L) arsenic concentration. A preliminary arsenic concentration in the tube well water of each household was tested with a rapid arsenic detection screening kit (Arsenic Low Range Test Kit, Hach, Loveland, CO, United States). A survey was completed by the mothers in each household. Stool samples from mothers and children and in-house drinking water samples were collected for microbiological analysis. The details of participants' enrollment and sample collection were described previously [60].

Determination of arsenic concentrations in water samples

Water samples (100 ml) from each household were sent to Eawag laboratory, Switzerland in sterile plastic bottles. Samples were analyzed with ICP-MS (Agilent-7500cx) for the quantification of arsenic compounds as described previously [61].

Enumeration and isolation of *E. coli*

Enumeration and isolation of total *E. coli*, third-generation cephalosporin-resistant (3GCr) *E. coli*, and fluoroquinolone-resistant (FQr) *E. coli* in each water and stool samples were done using the membrane filtration and drop plate methods, respectively, according to the procedures described previously [60]. For both water and stool samples, at least one isolated colony from each sample was confirmed as *E. coli* by API-20E (bioMérieux, France) and stored in glycerol broth at -80˚C for further characterization.

Antimicrobial susceptibility test

Antimicrobial susceptibility of *E. coli* (one isolate per sample) against 16 commercially available antibiotics (Oxoid Ltd., United Kingdom) was determined by standard disc diffusion technique following the Clinical and Laboratory Standards Institute (CLSI) guidelines and the procedure described previously [7]. *E. coli* ATCC 25922 was used as a control strain. An isolate resistant to at least one agent of three or more classes of antibiotics was considered multidrug-resistant (MDR) [62]. The presence of extended-spectrum β-lactamase (ESBL) in 3GCr *E. coli* was determined by the combination-disc diffusion (CDD) test following the CLSI guidelines [63]. A multiple antibiotic resistance (MAR) index was calculated based on the number of antibiotics one isolate was resistant to divided by the total number of antibiotics tested as described previously [64].

Test for susceptibility to arsenic

The minimum inhibitory concentrations (MICs) to sodium arsenite [As(III), $NaAsO_2$, Sigma] were determined by agar dilution assay performed according to the methods described previously [65]. Briefly, 10 μl of bacterial suspension (10^8 CFU/ml) from the overnight bacterial

culture was applied as a spot, in triplicate, on trypticase soy agar (TSA) plates supplemented with different concentrations of As(III) ranging from 0.5–11.0 mM. The plates were observed for MIC of arsenite salt after overnight incubation at 37˚C. The MIC was read as the lowest concentration with no visible growth. *E. coli* ATCC 25922 was used as the control strain. The isolates were considered resistant to arsenic if the As(III) MIC value was ≥5 mM (~1024 mg/L) [66].

PCR for ESBL and diarrheagenic genes among *E. coli* isolates

Boiled DNA extracted from *E. coli* isolates was used for the PCR assays. Primers specific for four major ESBL genes, $bla_{\text{CTX-M-1}}$, bla_{SHV}, $bla_{\text{TEM,}}$ and $bla_{\text{OXA-1}}$ were tested by PCR using primer sequences and PCR conditions as described previously [67]. Multiplex PCR was used for the detection of enteropathogenic *E. coli* (EPEC), enterotoxigenic *E. coli* (ETEC), enteroaggregative *E. coli* (EAEC), enteroinvasive *E. coli* (EIEC), and Shiga toxin-producing *E. coli* (STEC) according to the procedure described previously [9].

PCR for arsenic resistance genes

All *E. coli* isolates were tested for the presence of five major arsenic resistance genes, *arsA*, *arsB*, *arsC*, *arsD* and *arsR*. The primer sets and nucleotide sequences are presented in S2 Table. PCR reaction for *arsA*, *arsB*, and *arsC* was done according to the methods described previously [68]. Two different sets of primers were used to detect *arsR*; the K12_*arsR* primer set was used to detect chromosomally encoded *arsR* and the R46_*arsR* primer set was used to detect plasmid-encoded *arsR*. In addition, plasmid-encoded *arsD* was detected with another set of primers.

Whole genome sequence analysis

The whole genome sequencing (WGS) of 30 *E. coli* isolates was reported previously [60] and the data are available in GenBank under accession numbers CP050193-CP050222. These isolates were obtained from mother stool, child stool, and drinking water samples from an equal number of households in Matlab (n = 5) and Hajiganj (n = 5) areas based on their possible clonal linkage among isolates within the same household. Paired raw reads were trimmed using CLC Genomics Workbench v21.0.4 (https://digitalinsights.qiagen.com/) with a quality limit of 0.05, ambiguous nucleotides (max = 2), max length of 150 nucleotides, and a minimum length of 50 nucleotides. Reads were then mapped to reference sequence *E. coli* K-12 substr. MG1655 (Accession: NZ_CP032667.1). Ribosomal MLST (rMLST) sequences were indexed from 53 genes encoding the bacterial ribosome protein subunits (*rps* genes) using QC-checked reads [69]. The indexed *rps* genes were concatenated to construct the rMLST tree generated by R-package *ggtree v3.2.1* [70]. FASTA sequences containing *arsR*, *arsB*, and *arsC* genes were extracted from the mapped sequences and concatenated. MLST was generated from QC-checked paired reads using the MLST *v2.0* database (2021-10-18) [69]. Multiple sequence alignment of concatenated *arsRBC* operons was generated by R-package *MSA v1.26.0* using the ClustalW alignment [71]. Virulence genes were identified using QC raw reads with VirulenceFinder *v2.0* database (2020-5-29) [72–74]. Resistance genes were identified using QC raw paired reads with the ResFinder *v4.1* database (2022-02-04) and the PointFinder database (2021-02-01) [72,75,76]. Heatmaps were generated by R-package *ComplexHeatmap v2.10.0* using binary distances [77].

Statistical analyses

Data were entered in SPSS 20.0 (IBM Inc., Chicago, IL, United States). Data cleaning, statistical analyses, and graphical presentations were done in Stata 13.0 (College Station, TX, United

States), GraphPad Prism 9 (La Jolla, CA, United States), and R-3.4.2 (R Core Team, 2014). Chi-squared tests were used for the contingency tables where applicable (e.g., comparing the prevalence of *E. coli*, 3GCr *E. coli*, FQr *E. coli*, or arsenic resistance genes in each type of sample between two areas). The abundance of *E. coli*, 3GCr *E. coli*, and FQr *E. coli* in water and stool samples was calculated by $\log_{10}$ transformed colony counts (CFU/100 ml water or CFU/g stool) and the comparison between the two areas was done using the non-parametric Mann-Whitney test. Due to the high number of negative samples, only the positive samples were used in the analysis. The association between demographic characteristics of children and mothers in low versus high arsenic areas with a prevalence of fecal carriage of 3GCr *E. coli* and FQr *E. coli* was calculated using Chi-squared tests (Fisher's exact test was used if the expected frequency was <5). Familywise error rates accounted for multiple comparisons were adjusted using the Holm-Bonferroni test [78]. The odds ratios and their 95% confidence intervals (CIs) were estimated using the logistic regression with antibiotic resistance as a binary outcome and arsenic resistance as a binary exposure variable. A forest plot was used to present and compare the odds ratios among different antibiotics. Data were analyzed by R-package *glm v4.2.1*. Statistical significance was determined using $p < 0.05$ for all tests.

Supporting information

S1 Table. Arsenic concentration in water samples from Hajiganj and Matlab areas.
(DOCX)

S2 Table. PCR primers used in this study to identify arsenic resistance genes.
(DOCX)

S1 Fig. Antibiotic susceptibility of *E. coli* isolates. Antibiotic resistance of all *E. coli* isolates in A) drinking water, B) mother stool, and C) child stool samples collected from Hajiganj (high arsenic exposure) and Matlab (low arsenic exposure) was determined against 16 commercially available antibiotics as described in the Methods section. AMP, ampicillin; CN, gentamycin; TE, tetracycline; MEM, meropenem; IMP, imipenem; CRO, ceftriaxone, CTX, cefotaxime; CAZ, ceftazidime; FEP, cefepime; CT, colistin; CIP, ciprofloxacin; NA, nalidixic acid; AZM, azithromycin; SXT, trimethoprim-sulfamethoxazole; F, nitrofurantoin; C, chloramphenicol. 'n' indicates the number of *E. coli* isolates.
(TIFF)

S1 Data. Data set used to generate tables and figures.
(XLSX)

Acknowledgments

We thank Stephan Hug and Thomas Rüttimann from Eawag, Switzerland for measuring arsenic in water samples. The International Centre for Diarrhoeal Disease Research, Bangladesh (icddr,b) is thankful to the governments of Bangladesh, Canada, Sweden, and the United Kingdom for providing core/unrestricted support.

Author Contributions

Conceptualization: Timothy R. Julian, Mohammad Aminul Islam.

Data curation: Mohammed Badrul Amin, Prabhat Kumar Talukdar, Muhammad Asaduzzaman, Md. Rayhanul Islam, Mohammad Aminul Islam.

Formal analysis: Mohammed Badrul Amin, Prabhat Kumar Talukdar, Muhammad Asaduzzaman, Brandon M. Flatgard, Md. Rayhanul Islam, Yushuf Sharker, Mohammad Aminul Islam.

Funding acquisition: Tala Navab-Daneshmand, Molly L. Kile, Karen Levy, Timothy R. Julian, Mohammad Aminul Islam.

Methodology: Mohammed Badrul Amin, Prabhat Kumar Talukdar, Subarna Roy, Md. Rayhanul Islam, Sumita Rani Saha, Yushuf Sharker, Zahid Hayat Mahmud, Mohammad Aminul Islam.

Software: Yushuf Sharker.

Supervision: Mohammad Aminul Islam.

Writing – original draft: Mohammed Badrul Amin, Prabhat Kumar Talukdar, Muhammad Asaduzzaman, Yushuf Sharker, Mohammad Aminul Islam.

Writing – review & editing: Mohammed Badrul Amin, Prabhat Kumar Talukdar, Muhammad Asaduzzaman, Md. Rayhanul Islam, Zahid Hayat Mahmud, Tala Navab-Daneshmand, Molly L. Kile, Karen Levy, Timothy R. Julian, Mohammad Aminul Islam.

References

1. Antimicrobial Resistance C. Global burden of bacterial antimicrobial resistance in 2019: a systematic analysis. Lancet. 2022. Epub 20220118. https://doi.org/10.1016/S0140-6736(21)02724-0 PMID: 35065702.
2. Rousham EK, Asaduzzaman M, Mozmader T, Amin MB, Rahman M, Hossain MI, et al. Human colonization with extended-spectrum beta-lactamase-producing *E. coli* in relation to animal and environmental exposures in Bangladesh: an observational one health study. Environ Health Perspect. 2021; 129 (3):37001. Epub 20210303. https://doi.org/10.1289/EHP7670 PMID: 33656920; PubMed Central PMCID: PMC7929562.
3. Asaduzzaman M, Rousham E, Unicomb L, Islam MR, Amin MB, Rahman M, et al. Spatiotemporal distribution of antimicrobial resistant organisms in different water environments in urban and rural settings of Bangladesh. Sci Total Environ. 2022; 831:154890. Epub 20220329. https://doi.org/10.1016/j.scitotenv.2022.154890 PMID: 35364179.
4. Amin MB, Sraboni AS, Hossain MI, Roy S, Mozmader TAU, Unicomb L, et al. Occurrence and genetic characteristics of *mcr-1*-positive colistin-resistant *E. coli* from poultry environments in Bangladesh. J Glob Antimicrob Resist. 2020; 22:546–52. Epub 2020/04/29. https://doi.org/10.1016/j.jgar.2020.03.028 PMID: 32344122.
5. Montealegre MC, Talavera Rodriguez A, Roy S, Hossain MI, Islam MA, Lanza VF, et al. High genomic diversity and heterogenous origins of pathogenic and antibiotic-resistant *Escherichia coli* in household settings represent a challenge to reducing transmission in low-income settings. mSphere. 2020;5(1). Epub 20200115. https://doi.org/10.1128/mSphere.00704-19 PMID: 31941809; PubMed Central PMCID: PMC6968650.
6. Amin MB, Saha SR, Islam MR, Haider SMA, Hossain MI, Chowdhury A, et al. High prevalence of plasmid-mediated quinolone resistance (PMQR) among *E. coli* from aquatic environments in Bangladesh. PLoS One. 2021; 16(12):e0261970. Epub 20211229. https://doi.org/10.1371/journal.pone.0261970 PMID: 34965260; PubMed Central PMCID: PMC8716050.
7. Islam MA, Amin MB, Roy S, Asaduzzaman M, Islam MR, Navab-Daneshmand T, et al. Fecal colonization with multidrug-resistant *E. coli* among healthy infants in rural Bangladesh. Front Microbiol. 2019; 10:640. Epub 20190402. https://doi.org/10.3389/fmicb.2019.00640 PMID: 31001224; PubMed Central PMCID: PMC6454046.
8. Hasan B, Faruque R, Drobni M, Waldenstrom J, Sadique A, Ahmed KU, et al. High prevalence of antibiotic resistance in pathogenic *Escherichia coli* from large- and small-scale poultry farms in Bangladesh. Avian Dis. 2011; 55(4):689–92. Epub 2012/02/09. https://doi.org/10.1637/9686-021411-Reg.1 PMID: 22312993.
9. Talukdar PK, Rahman M, Rahman M, Nabi A, Islam Z, Hoque MM, et al. Antimicrobial resistance, virulence factors and genetic diversity of *Escherichia coli* isolates from household water supply in Dhaka,

Bangladesh. PLoS One. 2013; 8(4):e61090. Epub 2013/04/11. https://doi.org/10.1371/journal.pone.0061090 PMID: 23573295; PubMed Central PMCID: PMC3615999.

10. Rahman MM, Husna A, Elshabrawy HA, Alam J, Runa NY, Badruzzaman ATM, et al. Isolation and molecular characterization of multidrug-resistant *Escherichia coli* from chicken meat. Sci Rep. 2020; 10 (1):21999. Epub 2020/12/16. https://doi.org/10.1038/s41598-020-78367-2 PMID: 33319793; PubMed Central PMCID: PMC7738667.

11. Kohanski MA, DePristo MA, Collins JJ. Sublethal antibiotic treatment leads to multidrug resistance via radical-induced mutagenesis. Mol Cell. 2010; 37(3):311–20. https://doi.org/10.1016/j.molcel.2010.01.003 PMID: 20159551; PubMed Central PMCID: PMC2840266.

12. Huang L, Ahmed S, Gu Y, Huang J, An B, Wu C, et al. The effects of natural products and environmental conditions on antimicrobial resistance. Molecules. 2021; 26(14). Epub 20210714. https://doi.org/10.3390/molecules26144277 PMID: 34299552; PubMed Central PMCID: PMC8303546.

13. Zhu YG, Johnson TA, Su JQ, Qiao M, Guo GX, Stedtfeld RD, et al. Diverse and abundant antibiotic resistance genes in Chinese swine farms. Proc Natl Acad Sci U S A. 2013; 110(9):3435–40. Epub 20130211. https://doi.org/10.1073/pnas.1222743110 PMID: 23401528; PubMed Central PMCID: PMC3587239.

14. Guo X, Liu S, Wang Z, Zhang XX, Li M, Wu B. Metagenomic profiles and antibiotic resistance genes in gut microbiota of mice exposed to arsenic and iron. Chemosphere. 2014; 112:1–8. Epub 2014/07/23. https://doi.org/10.1016/j.chemosphere.2014.03.068 PMID: 25048881.

15. Chen S, Li X, Sun G, Zhang Y, Su J, Ye J. Heavy metal induced antibiotic resistance in bacterium LSJC7. Int J Mol Sci. 2015; 16(10):23390–404. Epub 2015/10/02. https://doi.org/10.3390/ijms161023390 PMID: 26426011; PubMed Central PMCID: PMC4632705.

16. Lemire JA, Harrison JJ, Turner RJ. Antimicrobial activity of metals: mechanisms, molecular targets and applications. Nat Rev Microbiol. 2013; 11(6):371–84. Epub 20130513. https://doi.org/10.1038/nrmicro3028 PMID: 23669886.

17. Tchounwou PB, Yedjou CG, Patlolla AK, Sutton DJ. Heavy metal toxicity and the environment. Exp Suppl. 2012; 101:133–64. https://doi.org/10.1007/978-3-7643-8340-4_6 PMID: 22945569; PubMed Central PMCID: PMC4144270.

18. Briffa J, Sinagra E, Blundell R. Heavy metal pollution in the environment and their toxicological effects on humans. Heliyon. 2020; 6(9):e04691. Epub 20200908. https://doi.org/10.1016/j.heliyon.2020.e04691 PMID: 32964150; PubMed Central PMCID: PMC7490536.

19. Cycon M, Mrozik A, Piotrowska-Seget Z. Antibiotics in the soil environment-degradation and their impact on microbial activity and diversity. Front Microbiol. 2019; 10:338. Epub 20190308. https://doi.org/10.3389/fmicb.2019.00338 PMID: 30906284; PubMed Central PMCID: PMC6418018.

20. Kuivenhoven M, Mason K. Arsenic toxicity. StatPearls. Treasure Island (FL)2022.

21. Shaji E, Santosh M, Sarath KV, Prakash P, Deepchand V, Divya BV. Arsenic contamination of groundwater: a global synopsis with focus on the Indian Peninsula. Geosci Front. 2021; 12(3):1–18. https://doi.org/10.1016/j.gsf.2020.08.015

22. Chung JY, Yu SD, Hong YS. Environmental source of arsenic exposure. J Prev Med Public Health. 2014; 47(5):253–7. Epub 2014/10/07. https://doi.org/10.3961/jpmph.14.036 PMID: 25284196; PubMed Central PMCID: PMC4186553.

23. Ahmad SA, Khan MH, Haque M. Arsenic contamination in groundwater in Bangladesh: implications and challenges for healthcare policy. Risk Manag Healthc Policy. 2018; 11:251–61. Epub 2018/12/26. https://doi.org/10.2147/RMHP.S153188 PMID: 30584381; PubMed Central PMCID: PMC6281155.

24. Chakraborti D, Rahman MM, Mukherjee A, Alauddin M, Hassan M, Dutta RN, et al. Groundwater arsenic contamination in Bangladesh-21 years of research. J Trace Elem Med Biol. 2015; 31:237–48. Epub 20150114. https://doi.org/10.1016/j.jtemb.2015.01.003 PMID: 25660323.

25. Islam MS, Mostafa MG. Influence of chemical fertilizers on arsenic mobilization in the alluvial Bengal delta plain: a critical review. J Water Supply Res Tech-Aqua 2021; 70(7):948–70.

26. Rahman M, Abdullah-Al-Mamun M, Khatun MS, Khan AS, Sarkar OS, Islam OK, et al. Contamination of selected toxic elements in integrated chicken-fish farm settings of Bangladesh and associated human health risk assessments. Biol Trace Elem Res. 2022. https://doi.org/10.1007/s12011-022-03244-0 PMID: 35449492

27. Uddin MM, Zakeel MCM, Zavahir JS, Marikar F, Jahan I. Heavy metal accumulation in rice and aquatic plants used as human food: a general review. Toxics. 2021; 9(12). Epub 20211220. https://doi.org/10.3390/toxics9120360 PMID: 34941794; PubMed Central PMCID: PMC8706345.

28. Rahaman MS, Rahman MM, Mise N, Sikder MT, Ichihara G, Uddin MK, et al. Environmental arsenic exposure and its contribution to human diseases, toxicity mechanism and management. Environ Pollut. 2021; 289:117940. Epub 20210810. https://doi.org/10.1016/j.envpol.2021.117940 PMID: 34426183.

29. Mori S, Lowenstein EJ, Steffen C. The largest mass poisoning in history: arsenic contamination of well water in Bangladesh. Skinmed. 2018; 16(4):265–7. Epub 20180701. PMID: 30207531.

30. Milton AH, Smith W, Rahman B, Hasan Z, Kulsum U, Dear K, et al. Chronic arsenic exposure and adverse pregnancy outcomes in Bangladesh. Epidemiology. 2005; 16(1):82–6. https://doi.org/10.1097/01.ede.0000147105.94041.e6 PMID: 15613949.

31. Wang W, Xie Z, Lin Y, Zhang D. Association of inorganic arsenic exposure with type 2 diabetes mellitus: a meta-analysis. J Epidemiol Community Health. 2014; 68(2):176–84. Epub 20131016. https://doi.org/10.1136/jech-2013-203114 PMID: 24133074.

32. Moon KA, Guallar E, Umans JG, Devereux RB, Best LG, Francesconi KA, et al. Association between exposure to low to moderate arsenic levels and incident cardiovascular disease. A prospective cohort study. Ann Intern Med. 2013; 159(10):649–59. https://doi.org/10.7326/0003-4819-159-10-201311190-00719 PMID: 24061511; PubMed Central PMCID: PMC4157936.

33. Rahman A, Vahter M, Ekstrom EC, Persson LA. Arsenic exposure in pregnancy increases the risk of lower respiratory tract infection and diarrhea during infancy in Bangladesh. Environ Health Perspect. 2011; 119(5):719–24. Epub 20101209. https://doi.org/10.1289/ehp.1002265 PMID: 21147604; PubMed Central PMCID: PMC3094427.

34. Dong X, Shulzhenko N, Lemaitre J, Greer RL, Peremyslova K, Quamruzzaman Q, et al. Arsenic exposure and intestinal microbiota in children from Sirajdikhan, Bangladesh. PLoS One. 2017; 12(12): e0188487. Epub 2017/12/07. https://doi.org/10.1371/journal.pone.0188487 PMID: 29211769; PubMed Central PMCID: PMC5718612.

35. Abbas SZ, Riaz M, Ramzan N, Zahid MT, Shakoori FR, Rafatullah M. Isolation and characterization of arsenic resistant bacteria from wastewater. Braz J Microbiol. 2014; 45(4):1309–15. Epub 20150304. https://doi.org/10.1590/s1517-83822014000400022 PMID: 25763035; PubMed Central PMCID: PMC4323304.

36. Maizel D, Balverdi P, Rosen B, Sales AM, Ferrero MA. Arsenic-hypertolerant and arsenic-reducing bacteria isolated from wells in Tucuman, Argentina. Can J Microbiol. 2018; 64(11):876–86. Epub 20180710. https://doi.org/10.1139/cjm-2017-0535 PMID: 29990443.

37. Dey U, Chatterjee S, Mondal NK. Isolation and characterization of arsenic-resistant bacteria and possible application in bioremediation. Biotechnol Rep (Amst). 2016; 10:1–7. Epub 20160215. https://doi.org/10.1016/j.btre.2016.02.002 PMID: 28352518; PubMed Central PMCID: PMC5040859.

38. Escalante G, Campos VL, Valenzuela C, Yanez J, Zaror C, Mondaca MA. Arsenic resistant bacteria isolated from arsenic contaminated river in the Atacama Desert (Chile). Bull Environ Contam Toxicol. 2009; 83(5):657–61. https://doi.org/10.1007/s00128-009-9868-4 PMID: 19779656.

39. Kumar P, Dash B, Suyal DC, Gupta SB, Singh AK, Chowdhury T, et al. Characterization of arsenic-resistant *Klebsiella pneumoniae* RnASA11 from contaminated soil and water samples and its bioremediation potential. Curr Microbiol. 2021; 78(8):3258–67. Epub 20210707. https://doi.org/10.1007/s00284-021-02602-w PMID: 34230990.

40. Sutterlin S, Tellez-Castillo CJ, Anselem L, Yin H, Bray JE, Maiden MCJ. Heavy metal susceptibility of *Escherichia coli* isolated from urine samples from Sweden, Germany, and Spain. Antimicrob Agents Chemother. 2018; 62(5). Epub 20180426. https://doi.org/10.1128/AAC.00209-18 PMID: 29530862; PubMed Central PMCID: PMC5923176.

41. Hobman JL, Crossman LC. Bacterial antimicrobial metal ion resistance. J Med Microbiol. 2015; 64(Pt 5):471–97. Epub 20141123. https://doi.org/10.1099/jmm.0.023036-0 PMID: 25418738.

42. Zhang M, Wan K, Zeng J, Lin W, Ye C, Yu X. Co-selection and stability of bacterial antibiotic resistance by arsenic pollution accidents in source water. Environ Int. 2020; 135:105351. Epub 20191130. https://doi.org/10.1016/j.envint.2019.105351 PMID: 31794937.

43. Cui L, Zhang YJ, Huang WE, Zhang BF, Martin FL, Li JY, et al. Surface-enhanced Raman spectroscopy for identification of heavy metal arsenic(V)-mediated enhancing effect on antibiotic resistance. Anal Chem. 2016; 88(6):3164–70. Epub 2016/02/18. https://doi.org/10.1021/acs.analchem.5b04490 PMID: 26885563.

44. Wang HT, Chi QQ, Zhu D, Li G, Ding J, An XL, et al. Arsenic and sulfamethoxazole increase the incidence of antibiotic resistance genes in the gut of earthworm. Environ Sci Technol. 2019; 53(17):10445–53. Epub 2019/08/03. https://doi.org/10.1021/acs.est.9b02277 PMID: 31373490.

45. Biswas M, Roy DN, Tajmim A, Rajib SS, Hossain M, Farzana F, et al. Prescription antibiotics for outpatients in Bangladesh: a cross-sectional health survey conducted in three cities. Ann Clin Microbiol Antimicrob. 2014; 13:15. Epub 20140422. https://doi.org/10.1186/1476-0711-13-15 PMID: 24755269; PubMed Central PMCID: PMC4004450.

46. Islam MA, Akhtar Z, Hassan MZ, Chowdhury S, Rashid MM, Aleem MA, et al. Pattern of antibiotic dispensing at pharmacies according to the WHO Access, Watch, Reserve (AWaRe) classification in

Bangladesh. Antibiotics (Basel). 2022; 11(2). Epub 20220214. https://doi.org/10.3390/antibiotics11020247 PMID: 35203851; PubMed Central PMCID: PMC8868217.

47. Frazee BW, Trivedi T, Montgomery M, Petrovic DF, Yamaji R, Riley L. Emergency department urinary tract infections caused by extended-spectrum beta-lactamase-producing Enterobacteriaceae: many patients have no identifiable risk factor and discordant empiric therapy is common. Ann Emerg Med. 2018; 72(4):449–56. Epub 20180703. https://doi.org/10.1016/j.annemergmed.2018.05.006 PMID: 29980462.

48. Manges AR, Perdreau-Remington F, Solberg O, Riley LW. Multidrug-resistant *Escherichia coli* clonal groups causing community-acquired bloodstream infections. J Infect. 2006; 53(1):25–9. Epub 20051102. https://doi.org/10.1016/j.jinf.2005.09.012 PMID: 16269177.

49. Adams-Sapper S, Diep BA, Perdreau-Remington F, Riley LW. Clonal composition and community clustering of drug-susceptible and -resistant *Escherichia coli* isolates from bloodstream infections. Antimicrob Agents Chemother. 2013; 57(1):490–7. Epub 20121112. https://doi.org/10.1128/AAC.01025-12 PMID: 23147723; PubMed Central PMCID: PMC3535975.

50. AsMat Final Report. Arsenic in tube well water and health consequences. Sida Project, Dhaka. 2007.

51. Kile ML, Houseman EA, Breton CV, Smith T, Quamruzzaman Q, Rahman M, et al. Dietary arsenic exposure in Bangladesh. Environ Health Perspect. 2007; 115(6):889–93. Epub 20070220. https://doi.org/10.1289/ehp.9462 PMID: 17589596; PubMed Central PMCID: PMC1892146.

52. Khan SI, Ahmed AK, Yunus M, Rahman M, Hore SK, Vahter M, et al. Arsenic and cadmium in food-chain in Bangladesh—an exploratory study. J Health Popul Nutr. 2010; 28(6):578–84. https://doi.org/10.3329/jhpn.v28i6.6606 PMID: 21261203; PubMed Central PMCID: PMC2995026.

53. Qi Z, Qi Y, Le Z, Han F, Li F, Yang H, et al. The interactions between antibiotic resistance genes and heavy metal pollution under co-selective pressure influenced the bio-enzyme activity. Front Chem. 2021; 9:691565. Epub 2021/08/03. https://doi.org/10.3389/fchem.2021.691565 PMID: 34336788; PubMed Central PMCID: PMC8316601.

54. Rosen BP. Families of arsenic transporters. Trends Microbiol. 1999; 7(5):207–12. https://doi.org/10.1016/s0966-842x(99)01494-8 PMID: 10354596.

55. Li AD, Li LG, Zhang T. Exploring antibiotic resistance genes and metal resistance genes in plasmid metagenomes from wastewater treatment plants. Front Microbiol. 2015; 6:1025. Epub 2015/10/07. https://doi.org/10.3389/fmicb.2015.01025 PMID: 26441947; PubMed Central PMCID: PMC4585309.

56. Zhang XZ, Lei CW, Zeng JX, Chen YP, Kang ZZ, Wang YL, et al. An IncX1 plasmid isolated from *Salmonella enterica* subsp. *enterica* serovar Pullorum carrying $bla_{\text{TEM-1B}}$, *sul2*, arsenic resistant operons. Plasmid. 2018; 100:14–21. Epub 2018/09/25. https://doi.org/10.1016/j.plasmid.2018.09.007 PMID: 30248363.

57. Kumar A, Rahman MS, Ali M, Salaun P, Gourain A, Kumar S, et al. Assessment of disease burden in the arsenic exposed population of Chapar village of Samastipur district, Bihar, India, and related mitigation initiative. Environ Sci Pollut Res Int. 2022; 29(18):27443–59. Epub 20220104. https://doi.org/10.1007/s11356-021-18207-6 PMID: 34982385.

58. Ahmed SM, Branscum A, Welch BM, Megowan M, Bethel JW, Odden MC, et al. A prospective cohort study of in utero and early childhood arsenic exposure and infectious disease in 4- to 5-year-old Bangladeshi children. Environ Epidemiol. 2020; 4(2):e086. Epub 20200409. https://doi.org/10.1097/EE9.0000000000000086 PMID: 32656486; PubMed Central PMCID: PMC7319226.

59. Rahman A, Granberg C, Persson LA. Early life arsenic exposure, infant and child growth, and morbidity: a systematic review. Arch Toxicol. 2017; 91(11):3459–67. Epub 20170914. https://doi.org/10.1007/s00204-017-2061-3 PMID: 28905217.

60. Amin MB, Hoque KI, Roy S, Saha SR, Islam MR, Julian TR, et al. Identifying the sources of intestinal colonization with extended-spectrum beta-lactamase-producing *Escherichia coli* in healthy infants in the community. Front Microbiol. 2022; 13:803043. Epub 20220331. https://doi.org/10.3389/fmicb.2022.803043 PMID: 35432268; PubMed Central PMCID: PMC9008759.

61. Neumann A, Kaegi R, Voegelin A, Hussam A, Munir AK, Hug SJ. Arsenic removal with composite iron matrix filters in Bangladesh: a field and laboratory study. Environ Sci Technol. 2013; 47(9):4544–54. Epub 20130408. https://doi.org/10.1021/es305176x PMID: 23647491.

62. Magiorakos AP, Srinivasan A, Carey RB, Carmeli Y, Falagas ME, Giske CG, et al. Multidrug-resistant, extensively drug-resistant and pandrug-resistant bacteria: an international expert proposal for interim standard definitions for acquired resistance. Clin Microbiol Infect. 2012; 18(3):268–81. Epub 20110727. https://doi.org/10.1111/j.1469-0691.2011.03570.x PMID: 21793988.

63. Patel JB. Performance standards for antimicrobial susceptibility testing: Clinical and Laboratory Standards Institute; 2017.

64. Krumperman PH. Multiple antibiotic resistance indexing of *Escherichia coli* to identify high-risk sources of fecal contamination of foods. Appl Environ Microbiol. 1983; 46(1):165–70. https://doi.org/10.1128/aem.46.1.165–170.1983 PMID: 6351743; PubMed Central PMCID: PMC239283.

65. Mallik S, Virdi JS, Johri AK. Proteomic analysis of arsenite—mediated multiple antibiotic resistance in *Yersinia enterocolitica* biovar 1A. J Basic Microbiol. 2012; 52(3):306–13. Epub 20110923. https://doi.org/10.1002/jobm.201100109 PMID: 21953586.

66. Pandey N, Bhatt R. Arsenic resistance and accumulation by two bacteria isolated from a natural arsenic contaminated site. J Basic Microbiol. 2015; 55(11):1275–86. Epub 20150619. https://doi.org/10.1002/jobm.201400723 PMID: 26095615.

67. Islam MA, Islam M, Hasan R, Hossain MI, Nabi A, Rahman M, et al. Environmental spread of New Delhi metallo-β-lactamase-1-producing multidrug-resistant bacteria in Dhaka, Bangladesh. Appl Environ Microbiol. 2017; 83(15):e00793–17. https://doi.org/10.1128/AEM.00793-17 PMID: 28526792

68. Saltikov CW, Olson BH. Homology of *Escherichia coli* R773 *arsA*, *arsB*, and *arsC* genes in arsenic-resistant bacteria isolated from raw sewage and arsenic-enriched creek waters. Appl Environ Microbiol. 2002; 68(1):280–8. Epub 2002/01/05. https://doi.org/10.1128/AEM.68.1.280–288.2002 PMID: 11772637; PubMed Central PMCID: PMC126541.

69. Jolley KA, Bray JE, Maiden MCJ. Open-access bacterial population genomics: BIGSdb software, the PubMLST.org website and their applications. Wellcome Open Res. 2018; 3:124. Epub 20180924. https://doi.org/10.12688/wellcomeopenres.14826.1 PMID: 30345391; PubMed Central PMCID: PMC6192448.

70. Yu G, Smith DK, Zhu H, Guan Y, Lam TT. ggtree: an R package for visualization and annotation of phylogenetic trees with their covariates and other associated data. Methods Ecol Evol. 2017; 8(1):28–36. https://doi.org/10.1111/2041-210X.12628

71. Bodenhofer U, Bonatesta E, Horejs-Kainrath C, Hochreiter S. msa: an R package for multiple sequence alignment. Bioinformatics. 2015; 31(24):3997–9. Epub 20150826. https://doi.org/10.1093/bioinformatics/btv494 PMID: 26315911.

72. Clausen P, Aarestrup FM, Lund O. Rapid and precise alignment of raw reads against redundant databases with KMA. BMC Bioinformatics. 2018; 19(1):307. Epub 20180829. https://doi.org/10.1186/s12859-018-2336-6 PMID: 30157759; PubMed Central PMCID: PMC6116485.

73. Joensen KG, Scheutz F, Lund O, Hasman H, Kaas RS, Nielsen EM, et al. Real-time whole-genome sequencing for routine typing, surveillance, and outbreak detection of verotoxigenic *Escherichia coli*. J Clin Microbiol. 2014; 52(5):1501–10. Epub 20140226. https://doi.org/10.1128/JCM.03617-13 PMID: 24574290; PubMed Central PMCID: PMC3993690.

74. Malberg Tetzschner AM, Johnson JR, Johnston BD, Lund O, Scheutz F. *In silico* genotyping of *Escherichia coli* isolates for extraintestinal virulence genes by use of whole-genome sequencing data. J Clin Microbiol. 2020;58(10). Epub 20200922. https://doi.org/10.1128/JCM.01269-20 PMID: 32669379; PubMed Central PMCID: PMC7512150.

75. Bortolaia V, Kaas RS, Ruppe E, Roberts MC, Schwarz S, Cattoir V, et al. ResFinder 4.0 for predictions of phenotypes from genotypes. J Antimicrob Chemother. 2020; 75(12):3491–500. https://doi.org/10.1093/jac/dkaa345 PMID: 32780112; PubMed Central PMCID: PMC7662176.

76. Zankari E, Allesoe R, Joensen KG, Cavaco LM, Lund O, Aarestrup FM. PointFinder: a novel web tool for WGS-based detection of antimicrobial resistance associated with chromosomal point mutations in bacterial pathogens. J Antimicrob Chemother. 2017; 72(10):2764–8. https://doi.org/10.1093/jac/dkx217 PMID: 29091202; PubMed Central PMCID: PMC5890747.

77. Gu Z, Eils R, Schlesner M. Complex heatmaps reveal patterns and correlations in multidimensional genomic data. Bioinformatics. 2016; 32(18):2847–9. Epub 20160520. https://doi.org/10.1093/bioinformatics/btw313 PMID: 27207943.

78. Giacalone M, Agata Z, Cozzucoli PC, Alibrandi A. Bonferroni-Holm and permutation tests to compare health data: methodological and applicative issues. BMC Med Res Methodol. 2018; 18(1):81. Epub 20180720. https://doi.org/10.1186/s12874-018-0540-8 PMID: 30029629; PubMed Central PMCID: PMC6054729.
